# Modelling and analysis of the complement system signalling pathways: roles of C3, C5a and pro-inflammatory cytokines in SARS-CoV-2 infection

**DOI:** 10.7717/peerj.15794

**Published:** 2023-09-20

**Authors:** Didar Murad, Rehan Zafar Paracha, Muhammad Tariq Saeed, Jamil Ahmad, Ammar Mushtaq, Maleeha Humayun

**Affiliations:** 1 School of Interdisciplinary Engineering and Sciences/Department of Sciences, National University of Science and Technology, Islamabad, Pakistan; 2 Department of Computer Science and Information Technology, University of Malakand, Chakdara, Malakand, Pakistan

**Keywords:** Qualitative modelling, René Thomas, Biological regulatory network (BRN), Model checker (NuSMV), SMBioNet, Complement system, C3, C5a, PICyts, SARS-CoV-2

## Abstract

The complement system is an essential part of innate immunity. It is activated by invading pathogens causing inflammation, opsonization, and lysis *via* complement anaphylatoxins, complement opsonin’s and membrane attack complex (MAC), respectively. However, in SARS-CoV-2 infection overactivation of complement system is causing cytokine storm leading to multiple organs damage. In this study, the René Thomas kinetic logic approach was used for the development of biological regulatory network (BRN) to model SARS-CoV-2 mediated complement system signalling pathways. Betweenness centrality analysis in cytoscape was adopted for the selection of the most biologically plausible states in state graph. Among the model results, in strongly connected components (SCCs) pro-inflammatory cytokines (PICyts) oscillatory behaviour between recurrent generation and downregulation was found as the main feature of SARS-CoV-2 infection. Diversion of trajectories from the SCCs leading toward hyper-inflammatory response was found in agreement with *in vivo* studies that overactive innate immunity response caused PICyts storm during SARS-CoV-2 infection. The complex of negative regulators FI, CR1 and DAF in the inhibition of complement peptide (C5a) and PICyts was found desirable to increase immune responses. In modelling role of MAC and PICyts in lowering of SARS-CoV-2 titre was found coherent with experimental studies. Intervention in upregulation of C5a and PICyts by C3 was found helpful in back-and-forth variation of signalling pattern linked with the levels of PICyts. Moreover, intervention in upregulation of PICyts by C5a was found productive in downregulation of all activating factors in the normal SCCs. However, the computational model predictions require experimental studies to be validated by exploring the activation role of C3 and C5a which could change levels of PICyts at various phases of SARS-CoV-2 infection.

## Introduction

The coronavirus disease 2019 (COVID-19) is caused by a novel coronavirus (CoV), also known as severe acute respiratory syndrome coronavirus-2 (SARS-CoV-2) that leads to major mortality and morbidity in all countries of the world (Merad & Martin, 2020). The SARS-CoV-2 pathogenic potential erupted with full force in a city of Wuhan, Hubei China in December 2019 (Platto et al., 2021). Globally, pandemic has caused over 6.9 million deaths with 766 million confirmed cases in a report published by World Health Organization (WHO) on May 21, 2023 (WHO, 2023). Global mortality, morbidity, and economic losses due to SARS-CoV-2 infection are higher as compared to the two-corona virus related epidemics (Bawazir et al., 2020; Abdelrahman, Li & Wang, 2020; Abdelghany et al., 2021; Tang et al., 2020). The SARS-CoV epidemic took place in mainland China in 2002 and affected 8,000 people with global mortality rate of 10% (Bawazir et al., 2020). Middle East respiratory syndrome (MERS)-CoV epidemic emerged in the Kingdom of Saudi Arabia in 2012 and affected 2,519 people with mortalities 866 at fatality rate of 34.4% worldwide (Bawazir et al., 2020). Even though death rate due to SARS-CoV-2 is low as compared to SARS-CoV and MERS-CoV, but its high-speed transmission dynamic and fatalities have been remained alarming. However, effective health improvement was observed through vaccination globally but SARS-CoV-2 viral mutations and new variants (Challen et al., 2021; Lauring & Malani, 2021; Sarwar et al., 2021) will remain a serious concern. SARS-CoV-2 have caused acute respiratory distress syndrome (ARDS), acute kidney injury (AKI) (Noris, Benigni & Remuzzi, 2020), cardiomyopathy (Mihalopoulos et al., 2020; Martinez-Rojas, Vega-Vega & Bobadilla, 2020) and neurological complications (Najjar et al., 2020).

Pro-inflammatory cytokines (PICyts) are a group of proteins which mediate immunomodulation and inflammatory responses. PICyts include granulocyte-colony stimulating factor (G-CSF), interferon gamma-induced protein 10 (IP10), monocyte chemoattractant protein 1 (MCP1), tumor necrosis factor alpha (TNF$\alpha$) and interleukin-2 (IL-2), IL-6, and IL-8 *etc*. (Mahmudpour et al., 2020; Chen et al., 2020). These are responsible for activation of inflammatory cells such as macrophages, neutrophils, and lymphocytes. Once macrophages are activated, immuno-regulatory molecules (anti-inflammatory cytokines) Type I interferons IFN$\alpha \backslash \beta$ are produced that control pro-inflammatory production (Ferreira et al., 2018; Zhang & An, 2007). Pro- and anti-inflammatory responses are mediated by complement anaphylatoxins (complement peptides) C3a and C5a, which, are inflammatory mediators and cause inflammation during COVID-19 by triggering cytokine storm (Shibabaw et al., 2020). In critical COVID-19 patients, Type I interferons IFN-$\alpha$ and IFN-$\beta$ are found downregulated. The relation between C5a and Type I interferons is indirect. C5a mediated inflammation response can suppress Type I interferons (Alosaimi et al., 2021; Mueller-Ortiz et al., 2017). High concentration levels of PICyts have been reported during SARS-CoV-2 infection (Alosaimi et al., 2021; Merad & Martin, 2020; García, 2020).

The complement system (CS) is composed of more than 60 proteins present in plasma and on cell surfaces (Zewde, 2019). The CS is an integral part of innate immunity, that plays pivotal role in the identification and elimination of invading pathogens (Zewde et al., 2016; Zewde & Morikis, 2018). Once triggered, the CS attains host immunity against pathogens by causing inflammatory response through PICyts mediators (*e.g*., C3a and C5a), opsonization *via* complement opsonins (*e.g*., C3b, C4b and C5b) and lysis by membrane attack complex (MAC; C5b-9) (Mahmudpour et al., 2020; Fletcher-Sandersjöö & Bellander, 2020; West, Kolev & Kemper, 2018, Zewde et al., 2021). The CS creates a bridge between innate and acquired immunity. In adaptive immunity, it helps in B and T cells stimulation, and antigen presentation (Zewde et al., 2021). Moreover, CS stimulates antibodies most likely immunoglobulin G (lgG) and lgM in humoral immunity (Hirsch et al., 2020). In severe SARS-CoV-2 infection, CS positive regulators are overactivated and negative regulators are downregulated. Some of the SARS-CoV-2 experimentally observed expression levels of CS entities are given in Table S1. Elevated production of cytokines termed as cytokines storm due to the overactivation of CS causes immune disorders categorized as autoimmune diseases (Liu, Sawalha & Lu, 2021; Galeotti & Bayry, 2020; Velikova & Georgiev, 2021) and multiple organs injuries (MOI) (Costela-Ruiz et al., 2020; Chouaki Benmansour, Carvelli & Vivier, 2021; Hojyo et al., 2020).

**Figure 1:**
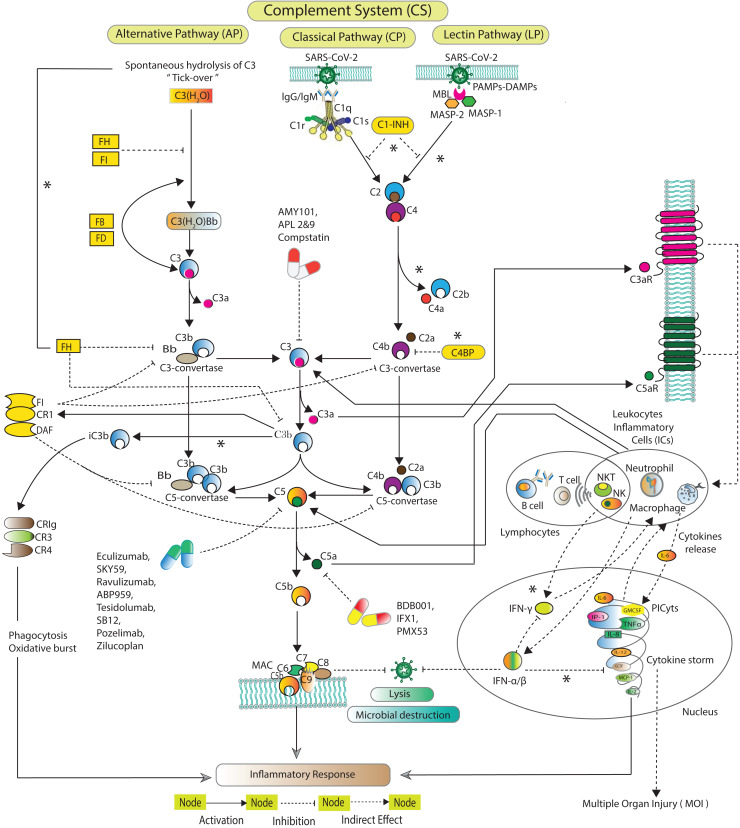
Schematic of the complement system activation and regulation. The SARS-CoV-2 induced complement system pathways, classical pathway (CP), lectin pathway (LP), and alternative pathway (AP) have been acquired from KEGG database and previous experimental studies (Shinjyo, Kagaya & Pekna, 2021; Detsika & Lianos, 2021; Dijkstra et al., 2019; Jodele & Köhl, 2021; Ng & Powell, 2021). The CP is initiated by C1q binds with the pathogen *via* antibodies IgG and/or IgM. The LP is activated by MBL-MASPs exposure to the PAMPs. The activation of AP is initiated due to tick-over known as spontaneous hydrolysis of C3 to C3(H_2_O). C3(H_2_O) binds with Bb a fragment of FB in the presence of FD formed pro-C3-convertase C3(H_2_O)Bb which produces C3b *via* cleavage of C3. The pro-C3-convertase is inhibited by FH and FI (Ng & Powell, 2021). In the fluid phase, C3b binds with Bb leading to the formation of C3-convertase C3bBb. C1s and MASPs cleaved C2 and C4 which are inhibited by C1-INH (Thomson et al., 2020; Bumiller-Bini et al., 2021; Ali et al., 2021). The cleaved fragments of C2 and C4 assembled to form C3-convertase C2aC4b. The C3-convertases are inhibited by C4BP, FH, FI, CR1 and DAF (Murugaiah et al., 2021). FH can inactivate C3b to iC3b binds with complement receptors CR3 (CD11b/CD18) and CR4 (CD11c/CD18). The formation of C5-convertases C3bBb3b and C4b2a3b are inhibited by FI, CR1 and DAF (Detsika & Lianos, 2021). The C5-convertases cleaved C5 into C5a and C5b. The potent anaphylatoxins C3a and C5a *via* C3aR and C5aR can produce PICyts which suppress the SARS-CoV-2 titre through the induction of inflammation response. The powerful opsonin C5b binds with C6 to C9 complements leading to the formation of terminal complement complex C5b-9 (MAC) which lysis the pathogen (Chouaki Benmansour, Carvelli & Vivier, 2021). C1-INH stands for complement component 1 esterase inhibitor; MBL, mannose binding lectin; MASP, MBL associated serine protease; FH, FI, FB and FD stand for factors H, I, B and D; DAF, decay accelerating factor; C3aR and C5aR, C3a and C5a receptors; PICyts, Pro-inflammatory cytokines; PAMPs, pathogen-associated molecular patterns. The components of CS that were not reduced to establish the BRN are marked with an asterisk (*).

The CS (Fig. 1) is activated *via* three distinct pathways classical pathway (CP), lectin pathway (LP), and alternative pathway (AP) (Zewde et al., 2016; Dunkelberger & Song, 2010; Bakshi et al., 2020; Lubbers et al., 2017). Each of the pathway has a unique initial activation mechanism. The CP is activated when C1q binds to antibody attached with antigen. C1q binds to pathogen surfaces and further activates C1r and C1s. The LP is activated when mannose binding lectin (MBL) attaches to a particular carbohydrate structure on microbes associated molecular patterns (MAMPs). MBL further triggering MBL associated serine proteases MASP-1 and MASP-2. Both CP and LP trigger consequences in cleavage of C2 and C4. The cleaved fragments form C3-convertase (C4bC2a), which, cleaves C3 into C3a and C3b. C3b covalently binds to microbial surface and make them more susceptible to phagocytosis. C4bC2a binds with C3b forming C5-convertase C4bC2aC3b. The AP is activated when C3 undergoes spontaneous hydrolysis to form initial C3-convertases C3(H_2_O)Bb. The AP involves Factors D (FD), which cleaves FB into Ba and Bb. Bb binds with C3b to make C3-convertase (C3bBb) that alternatively cleaves C3. C3bBb binds with the cleaved fragment C3b to eventually forms C5-convertase (C3bBbC3b). C5-convertases cleaves C5 to minor fragment C5a and major fragment C5b. C5a is the potent inflammatory complement peptide that produces massive PICyts (Mahmudpour et al., 2020). C5b combines with C6-C9 to make terminal complement complex (TCC) C5b-9 formation. The TCC can eliminate a pathogen by the lysis process (Chouaki Benmansour, Carvelli & Vivier, 2021).

**Figure 2:**
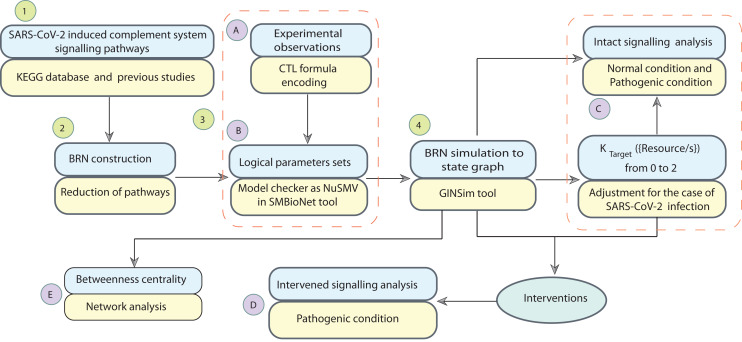
Workflow of the study. The computational modelling and analysis begin by exploring SARS-CoV-2 induced complement system signalling pathways from KEGG database and literature. This step is followed to construct the qualitative model BRN (Fig. 3). The reduction mechanism given in Table 1 is used for BRN construction. Formulated the computational tree logic (CTL) based on experimental observations. The CTL is used in the model checker NuSMV in SMBioNet tool for estimation of logical parameters sets. The model checker step-wise procedure is given in Fig. 4. The BRN is parameterized and simulated to the state graphs (Figs. 5–8) by using GINSim tool. The model is deployed for analysis of normal, pathogenic, and intervention signalling. The state graphs are rendered in cytoscape for further analysis of the signalling network, focused on maximum betweenness centrality of states.

**Figure 3:**
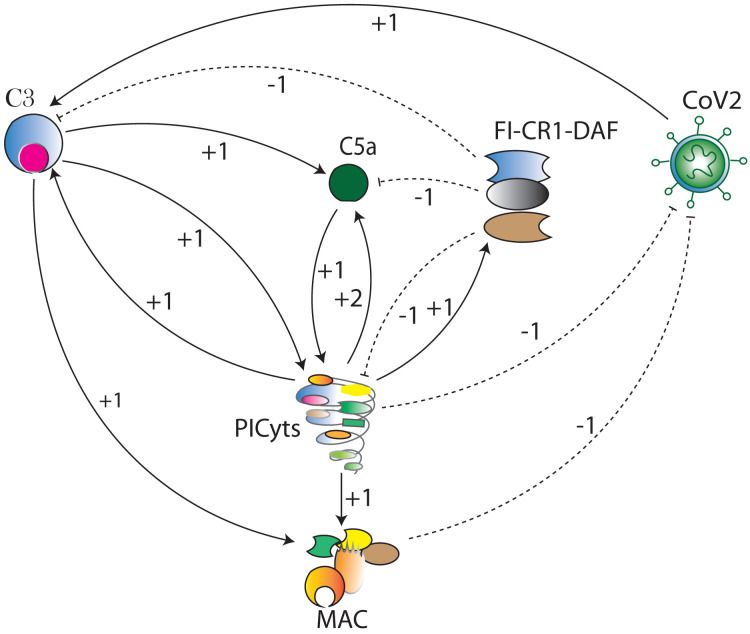
The BRN of complement system signalling pathways. The complement system signalling pathways (Fig. 1) are reduced to BRN. Using the reduction mechanism tabulated in Table 1 BRN is constructed. The edge (interaction) between resource and target entities is represented by arrowhead “ →” with a positive sign indicating activation (upregulation) and blunt edge “ ⊣” with a negative sign indicating inhibition (downregulation). The threshold levels of entities are represented by integers 1 and 2 on the edges (see definitions in File S1).

**Figure 4:**
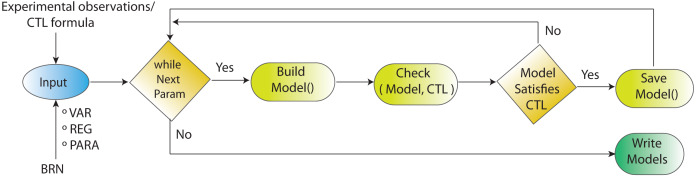
Workflow for parameters estimation using model checker. Model checking *via* NuSMV as model checker in SMBioNet tool is a sequential technique for the estimation of logical parameters (models) that completely enumerates through all achievable combinations of models. Model checker evaluates each model with respect to the CTL. If the outcome of model checker is true, the model is appended in the list of selected models.

Systems biology platform has been used for understanding the mechanism of SARS-CoV-2 interaction with host cells and it provides key insights into the effective molecular targets for the development of novel therapeutics (Hufsky et al., 2021; Rex et al., 2021). The study workflow is shown in Fig. 2. In this study, a qualitative model BRN (Fig. 3) was established for SARS-CoV-2 induced CS signalling pathways (Fig. 1) by using the René Thomas Kinetic logic approach. The advantage of qualitative modelling approach is that discrete model requires limited quantitative data for development of computation Tree Logic (CTL) formula to compute logical parameters (see workflow for parameters estimation in Fig. 4). To our knowledge, no computational kinetic logic model has previously been used to study CS signalling pathways until now for qualitative modelling of SARS-CoV-2 infection. The CS signalling pathways presented under various pathological condition of SARS-CoV-2 infection. The BRN includes different entities and transitions that implicate due to active repressing entities FI, CR1 and DAF complex, the upregulated signalling of C3, C5a and PICyts are repressed. C3 and PICyts mediated upregulation of MAC lowered titre of SARS-CoV-2. Due to upregulation of C3 and C5a PICyts over expressed. In therapeutic control strategies for the management of over production of PICyts an intervention modelling is performed. Intervening the overactive immune response, termed as deadlock state, can be removed and able to identify potential targets. Intervention in upregulation of C5a and PICyts due to C3 and upregulation of PICyts due to C5a diverted the system from hyper-inflammatory response to homeostatic responses. The study implies that targeted C3 in early-phase and C5a in late-phase could control expression level of PICyts, hyper-inflammation and ultimately SARS-CoV-2 infection. The computational results are biological sound and promising. The pieces of experimental information from previous studies agree with the results but require to be further validated in wet-laboratory experiment.

**Table 1:** The reduction mechanism of complement system (CS) pathways. The entities interaction is indicated by arrowhead “ →” for activation and blunt edge “ ⊣” for inhibition. The expression relations are indicated by “ *node*→*node*→*node*” inferred expression and “ *node*→*node*⊣*node*” inferred repression. The components of CS that were not reduced are marked with “ ∗” (see in Fig. 1).

S. No.	Pathways	Reduction
1	CoV2 $\to$ C1qrs $\to$ C2 and C4 complex $\to$ C3-convertase (C4b and C2a complex) $\to$ C3	CoV2 $\to$ C3
2	C3 $\to$ C3b $\to$ C5-convertase $\to$ C5 $\to$ C5a	C3 $\to$ C5a
3	C3 $\to$ C3b $\to$ C5-convertase $\to$ C5 $\to$ C5b $\to$ MAC	C3 $\to$ MAC
4	FI-CR1-DAF $\dashv$ C3-convertase $\to$ C3	FI-CR1-DAF $\dashv$ C3
5	FI-CR1-DAF $\dashv$ C5-convertase $\to$ C5 $\to$ C5a	FI-CR1-DAF $\dashv$ C5a
6	C3 $\to$ C3a $\to$ C3aR $\to$ ICs $\to$ PICyts	C3 $\to$ PICyts
7	PICyts $\to$ ICs $\to$ C3	PICyts $\to$ C3
8	C5a $\to$ C5aR $\to$ ICs $\to$ PICyts	C5a $\to$ PICyts
9	PICyts $\to$ ICs $\to$ C5 $\to$ C5a	PICyts $\to$ C5a
10	FI-CR1-DAF $\dashv$ C5-convertase $\to$ C5 $\to$ C5a $\to$ C5aR $\to$ ICs $\to$ PICyts or FI-CR1-DAF $\dashv$ C3-convertase $\to$ C3 $\to$ C3a $\to$ C3aR $\to$ ICs $\to$ PICyts	FI-CR1-DAF $\dashv$ PICyts
11	PICyts $\to$ ICs $\to$ C3 $\to$ C3b $\to$ FI-CR1-DAF	PICyts $\to$ FI-CR1-DAF
12	PICyts $\to$ ICs $\to$ C5 $\to$ C5b $\to$ MAC	PICyts $\to$ MAC
13	PICyts $\to$ ICs $\to$ IFN $\alpha \backslash \beta$ $\dashv$ CoV2	PICyts $\dashv$ CoV2
14	MAC $\dashv$ CoV2	MAC $\dashv$ CoV2

**Figure 5:**
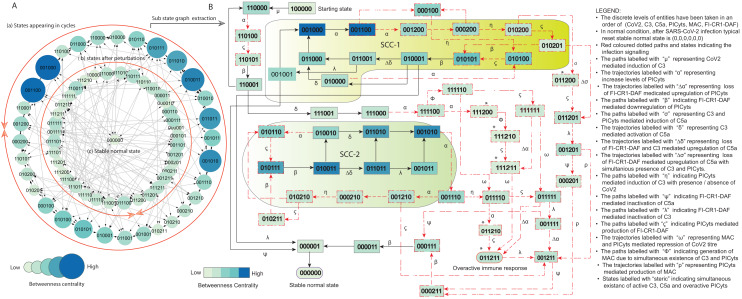
State graph and extracted sub state graph of complement system signalling during normal condition. (A) The state graph has been rendered comprising of 69 states and 190 trajectories. Within the states, integer values 0, 1, and 2 denoting inactive, active, and overactive levels of entities that are ordered in (CoV2, C3, C5a, PICyts, MAC, FI-CR1-DAF) fashion. The states are sorted by sizing and colouring of each state based on betweenness centrality (BC). Circular representation of the state graph comprises (a); the outermost circular shape indicates states appearing in periodic circles. (b) After perturbations, the trajectories from bifurcation states diverge to several states. Most of the trajectories converge to overactive immune response $(0,1,1,2,1,1)$ ; (c) eventually the paths lead to an attractor successful immune response $(0,0,0,0,0,0)$ termed as stable normal state, it BC is low as compared to other states. (B) Sub state graph is extracted from the main state graph that comprises stable normal state $(0,0,0,0,0,0)$ , homeostatic behaviours know as strongly connected components (SCC-1 and SCC-2). Paths indicate states transitions related to distinct signalling events are labelled with Greek letters (see in B).

**Figure 6:**
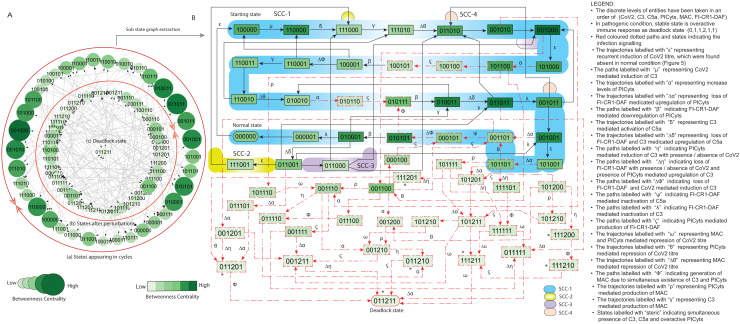
State graph and extracted sub state graph of complement system signalling during pathogenic condition. (A) State graph has been rendered comprising of 78 states and 229 trajectories. Within the states, integer values 0, 1, and 2 denoting inactive, active, and overactive levels of entities that are ordered in (CoV2, C3, C5a, PICyts, MAC, FI-CR1-DAF) fashion. The states are sorted by sizing and colouring of each state based on betweenness centrality (BC). Circular representation of state graph comprises of (a) outer most circular shape indicates states appearing in periodic circles. (b) After perturbations, the trajectories from bifurcation states diverge to several states (c) eventually the trajectories lead to an attractor as overactive immune response $(0,1,1,2,1,1)$ termed as deadlock state, it BC is low as compared to other states. (B) Sub state graph extracted from the main state graph that comprises deadlock state $(0,1,1,2,1,1)$ , homeostatic behaviours SCC-1 to SCC-4. Trajectories indicate states transitions related to distinct signalling events are labelled with Greek letters (see in B).

**Figure 7:**
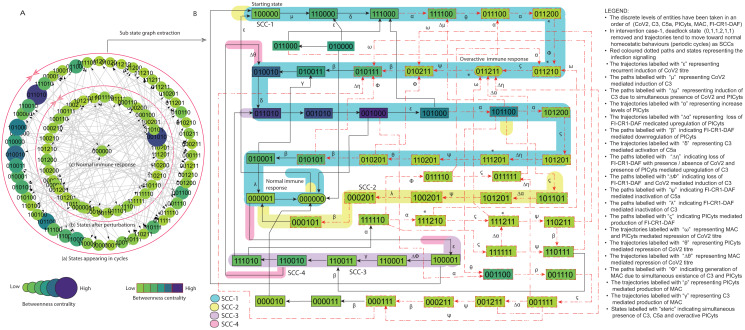
State graph and extracted sub state graph of complement system signalling for the case-1 (intervention in C3 mediated upregulation of C5a and PICyts). (A) State graph has been rendered comprising of 87 number of nodes and 263 trajectories. Within the states, integer values 0, 1, and 2 denoting inactive, active, and overactive levels of entities that are ordered in (CoV2, C3, C5a, PICyts, MAC, FI-CR1-DAF) fashion. The states are sorted by sizing and colouring of each state based on betweenness centrality (BC). Circular representation of state graph comprises (a) outer most circular shape represents states appearing in homeostatic behaviours. (b) After perturbations, the paths diverge to several states and eventually the state transitions continued in periodic circles that might be due to homeostatic control mechanism. (B) The sub state graph extracted from the main state graph that comprises homeostatic responses SCC-1 to SCC-4. Paths indicate states transitions related to distinct signalling events are labelled with Greek letters (see in the Figure B legend).

**Figure 8:**
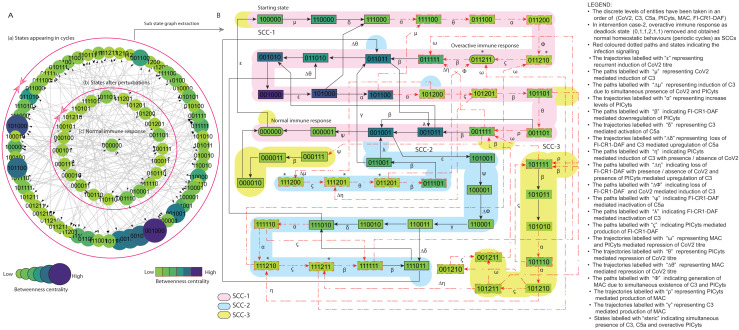
State graph and extracted sub state graph of complement system signalling for the case-2 (intervention in C5a mediated upregulation of PICyts). (A) State graph has been rendered comprising of 80 number of nodes and 240 trajectories. Within the states, integer values 0, 1, and 2 denoting inactive, active, and overactive levels of entities that are ordered in (CoV2, C3, C5a, PICyts, MAC, FI-CR1-DAF) fashion. The states are sorted by sizing and colouring of each state on the basis of betweenness centrality (BC). Circular represention of state graph comprises (a) outer most circular shape represents states appearing in periodic circles. (b) After perturbations, the paths diverge to several states and eventually that continued in periodic circles. (B) The substate graph extracted from the main state graph that comprises of homeostatic behaviours SCC-1 to SCC-3. Trajectories indicate states transitions related to distinct signalling events are labelled with Greek letters (see B).

## Materials and Methods

The qualitative (discrete) model of a biological regulatory network (BRN) represented as a labeled directed graph is based on the asynchronous Boolean logic approach of René Thomas (Thomas, 1991; Paracha et al., 2014; Saeed et al., 2018, 2016; Bibi et al., 2017; Aslam et al., 2014). Thomas identified limitations in the asynchronous Boolean logic as it deals with discrete values 0 or 1. Thomas generalized the asynchronous Boolean logic to an asynchronous multi-valued Kinetic logic which deals with more complex biological regulatory networks (Thomas & Kaufman, 2001). In this study, the kinetic logic approach was used to model SARS-CoV-2 induced complement system (CS) pathways activation and regulation.

The semantics of asynchronous multi-valued kinetic logic (Paracha et al., 2014; Paracha, 2015) and Boolean logic (Saeed et al., 2018, 2016; Bibi et al., 2017; Aslam et al., 2014) have been presented. The asynchronous Boolean logic is explained by considering a dummy BRN composed of three entities in the Supplemental File (File S1).

### Reduction of complement system signalling pathways

In this study, the CS signalling pathways are reduced to BRN. The reduction process is in accordance with several other studies (Paracha et al., 2014; Saeed et al., 2018, 2016; Bibi et al., 2017; Aslam et al., 2014). In reduction of pathways, the prominent entities of interest in the pathways are retained and abstracted the remaining, preserved the behaviours of removed entities. The components of CS that were not reduced and excluded in BRN development are marked with an asterisk (*) (see in Fig. 1). The reduction mechanism is given in Table 1. The BRN comprises of six entities CoV2, C3, C5a, PICyts, MAC, and FI-CR1-DAF.

The limitation of the kinetic logic approach is that it is used to analyze small networks (Paracha et al., 2014) due to its scalability limitation. For example, the CS signalling pathways (Fig. 1) are large networks and on simulation result in state graph would be composed of massive states. While, BRN (Fig. 3) established from CS pathways is a small network and on simulation gives reasonable number of states in state graphs shown in Figs. 5–8. For example, the state graph (Fig. 5) comprises of 69 states and 190 trajectories.

In different scientific studies signalling pathways are reduced to BRNs. An example of pathway consist of 13 entities reduced to BRN comprises of three entities as given in Figure 3.3 of (Saadatpour, Albert & Reluga, 2013) study. Similarly, pathway consist of 22 entities reduced to BRN involves of five entities as shown in Figure 2 of (Paracha et al., 2014) study. Additionally, some examples of dummy networks are available in Fig. S1.

### Computational tree logic development

Computational tree logic (CTL) is a branching time logic, means CTL model of time is a tree like structure in which the future is not set; there are various trajectories in the future, any one of which can be a real trajectory that is realized. CTL is applicable to the state graph of BRN for the exploration of system properties such as stable states and cycles. Temporal logic CTL is used to express the temporal property of the system. Biological system is non-deterministic, and in this respect, CTL is the most suitable due to it branching nature (Bernot et al., 2004). Fundamental idea for CTL formalism is given in File S2. In literature, different scientific studies are performed for qualitative modelling and analysis of diseases like sepsis, cancer, and dengue, respectively. The experimental observations are encoded as CTL formula (Paracha et al., 2014; Saeed et al., 2018, 2016; Bibi et al., 2017; Aslam et al., 2014). In this study, the SARS-CoV-2 experimental observations (Table S1) are encoded as CTL formula given in Eq. (1).

(1) $$\matrix{ {{\Psi _\alpha } = {\phi _1} \to EF({\phi _2})} \cr  {{\Psi _\beta } = {\phi _1} \to EF(AG({\phi _3}))} \cr  {{\Psi _\gamma } = {\phi _4} \to EX(EF({\phi _4}))} \cr  {\Psi = {\Psi _\alpha } \wedge {\Psi _\beta } \wedge {\Psi _\gamma }} \cr  }$$

The formula $\Psi$ comprises main quantifier and Boolean operators. The path quantifier “*A*” represents “ *along* *all* *paths*” implies the stated characteristics must hold in all the trajectories arise from the specified state. The existential quantifier as “*E*” indicates “ *along* *at* *least* *one* *path* (*there* *exist*)” implies the stated characteristics should hold in at least one trajectory from the specified state. The future quantifier as “*F*” indicates “ *some* *future* *state*” implies the stated characteristics should hold in one of the future states in the trajectory starting from the specified state. The next quantifier as “*X*” represents “ *next* *state*” implies the stated characteristics should hold in the sudden incomer state. The global quantifier as “*G*” indicates “ *all* *future* *states* (*globally*)” implies the stated characteristics hold in all states of a trajectory starting from specified state. Symbol “ →” indicates the Boolean implication operator and “ ∧” represents “ *and*” operator.

In the sub-formula ${\Psi _\alpha }$ , ${\phi _1}$ = (CoV2 = 1 $\wedge$ C3 = 0 $\wedge$ C5a = 0 $\wedge$ PICyts = 0 $\wedge$ MAC = 0 $\wedge$ FI-CR1-DAF = 0) represents an initial state in which the pathogen CoV2 present and all other entities are inactive. ${\phi _2}$ = (CoV2 = 1 $\wedge$ C3 = 1 $\wedge$ C5a = 1 $\wedge$ PICyts = 1) represents a future state in which positive regulators C3, C5a and PICyts are active. ${\Psi _\alpha }$ as first characteristic inferred the dynamic behaviours of the BRN in which trajectories from initial state leading toward overactive immune response termed as deadlock state. The infectious state as an attractor is the network future state.

In sub-formula ${\Psi _\beta }$ , followed initial state ${\phi _1}$ the future state is ${\phi _3}$ = (CoV2 = 0 $\wedge$ C3 = 0 $\wedge$ C5a = 0 $\wedge$ PICyts = 0 $\wedge$ MAC = 0 $\wedge$ FI-CR1-DAF = 0) in which all entities remain inactive. ${\Psi _\beta }$ as second characteristic implies successful immune response and all the entities downregulated. The normal state as an attractor is the system future state.

The third characteristic given as sub-formula ${\Psi _\gamma }$ reflect that starting from the state ${\phi _4}$ = (CoV2 = 0 $\wedge$ C3 = 1 $\wedge$ C5a = 1 $\wedge$ PICyts = 0 $\wedge$ MAC = 0 $\wedge$ FI-CR1-DAF = 0) the paths with cyclic behaviour going back to it previous original position for maintenance of immune system homeostasis and recurrent induction of initial entities implies persistence SARS-CoV2 infection.

### Model checking and logical parameters inference

The workflow for model checking *via* model checker NuSMV in SMBioNet (Selection of Models of Biological Networks) tool is shown in Fig. 4. Model checking is a classical method used for the evaluation of the BRN (Fig. 3) with governing CTL is given in Eq. (1).

For normal condition logical parameters sets with detail description of an input is given in File S3. Moreover, for pathogenic condition and intervention cases logical parameters sets are given in Files S7, S10, and S13. The parameters sets are depicted *via* heatmaps in Figs. S2, S5, S7, and S9.

Logical parameters in the BRN (Fig. 3) are represented by the equation ${K_{Target}}(\{ Resource/s\} ) = n \in {Z^ + }$ . The resource entity may be activator or inhibitor of the targeted entity. Activator is considered as resource when it is present, inhibitor/inactivator is considered as resource when it is absent. For example, PICyts and MAC are inhibitors of CoV2, ${K_{CoV2}}\{ \} = 0$ denotes MAC and PICyts as resource are active for inhibition of CoV2. ${K_{CoV2}}\{ MAC\} = 0$ denotes MAC is inactive and PICyts is active for inhibition of CoV2. Moreover, C3 and PICyts are activators and FI-CR1-DAF inhibitor of C5a, ${K_{C5a}}\{ C3\} = 1$ denotes C3 and FI-CR1-DAF are active and PICyts is inactive for C5a. Similarly for each entities logical parameters have been described in File S16.

### Network analysis

For investigation of important aspects and properties of the dynamical biological system in systems biology graph theory betweenness centrality (BC) analysis can be used. The BC helpful for identification of necessary states and significant SCCs in state graph (Saeed et al., 2016). A state with maximum BC indicates higher probability of its occurrence (Aslam et al., 2014). The BC analysis is instrumental in collection of biological meaningful and beneficial information about function of the system signalling pathways and identification of targets for drug discovery (Saeed et al., 2018). The mathematical definition of BC is given in definition 7 (File S1).

## Results

The SARS-CoV-2 induced complement system (CS) signalling was studied by considering normal and pathogenic conditions. In normal condition, the entities of the system get activated due to the presence of pathogen. The system ultimately leads to normal immune response termed as recovery/normal state $(0,0,0,0,0,0)$ (Fig. 5). Infection severity can be determined by up/downregulation of the entities in a specific pattern. In pathogenic condition, due to the persistence presence of pathogen (recurrent infection), the system tends to move toward overactive immune response termed as hyper-inflammatory stable state $(0,1,1,2,1,1)$ (Fig. 6). In both conditions, immune system perform homeostatic response or behavior (periodic cycle) represented in the state graph by strongly connected components (SCC) in which oscillatory behaviours of entities can be observed. Intervention was performed to model intervened signalling with the focus to eliminate deadlock state in pathogenic condition and deviation of trajectories (state transitions) toward SCCs as depicted in Figs. 7 and 8.

The state graph comprises of states with entities taken in an order of CoV2, C3, C5a, PICyts, MAC, and FI-CR1-DAF. The discrete values 0, 1, and 2 represent inactive, active, and overactive levels of entities, respectively. State transitions depend on logical parameters and threshold levels. Necessary states have been identified by using betweenness centrality (BC) analysis performed in cytoscope. The state graph representation in circular fashion comprises of states appearing in cycles placed on the outer most circular shape. States after perturbations placed on the inner circular shape as shown in Figs. 5A and 6A for normal and pathogenic conditions, respectively. Focusing on maximum BC of states, trajectories and SCCs to analysis the BRN dynamics in convenient way sub state graphs have been extracted from the main state graphs shown in Figs. 5B and 6B, respectively.

### Signalling in normal condition

The CTL given in Eq. (1) governed SMBioNet to generate parameters sets (models) are provided in File S3. The models have also been presented in the form of heatmap that represents the qualitative levels used in this study (Fig. S2). Selected set of logical parameters is tabulated in Table 2 and depicted *via* dummy tendency graphs (Fig. S3). Each logical parameter for the entities was devised based on the experimental studies reported in literature. The details and justifications for each logical parameter are described in File S4. For example, due to the presence of SARS-CoV-2, the concentration level of C5a increase that mediates the expression of PICyts and ultimately the generation of cytokines storm (Mahmudpour et al., 2020; Chouaki Benmansour, Carvelli & Vivier, 2021). Logical parameter devised as ${K_{PICyts}}(\{ C5a\} ) = 2$ indicates that PICyts are activated to a qualitative level “ 2” because of the presence of C5a (Alosaimi et al., 2021).

**Table 2:** The logical parameters set for modelling normal condition signalling. The logical parameters set indicates Model-14 is obtained from logical parameters sets (models) are provided in File S3. The BRN (GINSim File S5) for normal condition signalling is parameterized by using these parameters. The logical parameters are discussed in detail with experimental evidence (File S4).

S. No.	Logical parameters
1	${K_{CoV2}}\{ \} = 0$
2	${K_{CoV2}}\{ MAC\}$ = 0
3	${K_{CoV2}}\{ PICyts\}$ = 0
4	${K_{CoV2}}\{ MAC,PICyts\}$ = 0
5	${K_{C3}}\{ \}$ = 0
6	${K_{C3}}\{ CoV2\}$ = 1
7	${K_{C3}}\{ FI - CR1 - DAF\}$ = 0
8	${K_{C3}}\{ PICyts\}$ = 0
9	${K_{C3}}\{ CoV2,FI - CR1 - DAF\}$ = 1
10	${K_{C3}}\{ FI - CR1 - DAF,PICyts\}$ = 1
11	${K_{C3}}\{ CoV2,PICyts\}$ = 1
12	${K_{C3}}\{ CoV2,FI - CR1 - DAF,PICyts\}$ = 1
13	${K_{C5a}}\{ \}$ = 0
14	${K_{C5a}}\{ C3\}$ = 1
15	${K_{C5a}}\{ FI - CR1 - DAF\}$ = 0
16	${K_{C5a}}\{ PICyts\}$ = 0
17	${K_{C5a}}\{ C3,FI - CR1 - DAF\}$ = 1
18	${K_{C5a}}\{ FI - CR1 - DAF,PICyts\}$ = 0
19	${K_{C5a}}\{ C3,PICyts\}$ = 1
20	${K_{C5a}}\{ C3,FI - CR1 - DAF,PICyts\}$ = 1
21	${K_{MAC}}\{ \}$ = 0
22	${K_{MAC}}\{ C3\}$ = 1
23	${K_{MAC}}\{ PICyts\}$ = 1
24	${K_{MAC}}\{ C3,PICyts\}$ = 1
25	${K_{FI - CR1 - DAF}}\{ \}$ = 0
26	${K_{FI - CR1 - DAF}}\{ PICyts\}$ = 1
27	${K_{PICyts}}\{ \}$ = 0
28	${K_{PICyts}}\{ C3\}$ = 0
29	${K_{PICyts}}\{ C5a\}$ = 2
30	${K_{PICyts}}\{ FI - CR1 - DAF\}$ = 0
31	${K_{PICyts}}\{ C3,C5a\}$ = 2
32	${K_{PICyts}}\{ C5a,FI - CR1 - DAF\}$ = 2
33	${K_{PICyts}}\{ C3,FI - CR1 - DAF\}$ = 1
34	${K_{PICyts}}\{ C3,C5a,FI - CR1 - DAF\}$ = 2

The system has been modeled in the form of a BRN shown in Fig. 3 (GINSim File S5) that is parameterized by using logical parameters given in Table 2. The results were obtained as state graph given in GINSim (File S6) that is visualized in graphviz (Fig. S4). The normal condition signalling of different entities depicted in Fig. 5 is further used for interpretation.

Each logical parameter was taken in such a way that during normal condition, the immune system could perform homeostatic response (SCCs). SCCs led towards hyper-inflammatory state $(0,1,1,2,1,1)$ that ultimately converges to stable normal state $(0,0,0,0,0,0)$ shown in Fig. 5B.

Presence of SARS-CoV-2 in the system is represented by state $(1,0,0,0,0,0)$ , that upregulates C3 $(1,0,0,0,0,0)$ $\to$ $(1,1,0,0,0,0)$ labelled with “ *μ*” and initiate the rest of the complement system. The state $(1,0,0,0,0,0)$ is taken as the first state from which all the responses are generated. In recent experimental study of SARS-CoV-2 infection, inflammation is caused due to active C3 (Mastaglio et al., 2020). The path labeled with “ *α*” $(1,1,0,0,0)$ $\to$ $(1,1,0,1,0)$ represents C3 mediated induction of PICyts in the absence of inhibitory complex FI-CR1-DAF.

It has been reported that overactivated C5a can cause cytokine storm during the severe infection caused by SARS-CoV2 (Mahmudpour et al., 2020). The concentration level of C5a has also been correlated with the infection severity (Chouaki Benmansour, Carvelli & Vivier, 2021). Trajectories labeled with “ *α*” indicate increasing level of PICyts. C5a mediated induction of PICyts were found in trajectory $(0,0,1,0,0,0)$ $\to$ $(0,0,1,1,0,0)$ . Most importantly, over expressed PICyts were found due to C3 and/or C5a in the absence of FI-CR1-DAF in trajectories $(0,0,1,1,0,0)$ $\to$ $(0,0,1,2,0,0)$ , $(0,0,1,1,1,0)$ $\to$ $(0,0,1,2,1,0)$ , and $(0,1,1,1,1,0)$ $\to$ $(0,1,1,2,1,0)$ .

Indirect inhibitory role of FI-CR1-DAF of PICyts *via* C5a-C5aR (C5a receptor) and/or C3a-C3aR (Fig. 1) were found necessary in suppressing concentration level of PICyts and causing homeostatic response represented as (SCC-1 and SCC-2; with maximum betweenness centrality (BC)). Downregulation of PICyts due to the presence of FI-CR1-DAF are shown in trajectories labelled with “ *β*” , $(0,1,0,2,0,1)$ $\to$ $(0,1,0,1,0,1)$ $\to$ $(0,1,0,0,0,1)$ in SCC-1 and $(0,1,0,1,1,1)$ $\to$ $(0,1,0,0,1,1)$ in SCC-2. Trajectories from bifurcation states, for example $(0,1,0,2,0,0)$ , $(0,1,0,2,0,1)$ , $(0,0,1,2,1,0)$ , $(0,0,1,1,1,0)$ , $(0,0,1,0,0,1)$ , $(0,1,0,0,0,1)$ , and $(1,1,0,0,0,1)$ diverged to several states particularly linked to hyper-inflammatory states. Some trajectories lead to overactive immune response $(0,1,1,2,1,1)$ and ultimately converged to stable normal state $(0,0,0,0,0,0)$ .

In SARS-CoV-2 infection over activated inflammatory cells like macrophages and neutrophils cause over expressed PICyts (cytokine storm). In the CS signalling pathway, there is positive feedback loop between the inflammatory cells and PICyts (see CS signalling pathways in Fig. 1). C5a highly expressed due to the inflammatory cells. Activation of C5a was found due to C3 and PICyts as depicted in paths labelled with “ *σ*” . In feedback mechanism, PICyts can also activate C3. Paths labelled with “ *η*” represent activation of C3. C3 mediated activation of C5a are shown in trajectories labelled with “ *δ*” . Moreover, C5a was found upregulated in the absence of FI-CR1-DAF and presence of C3 and/or PICyts as depicted in paths labelled with “ *Δσ*” and “ *Δδ*” , respectively.

Simultaneous existence of active C3, C3a and overactive PICyts is shown in states labelled with an asterisk (*) (Fig. 5). This behaviour leads the system towards overactivated immune response. FI-CR1-DAF mediated downregulation of C3 and C5a is depicted in transitions labelled with “ *ψ*” and “ *λ*” , respectively. This inhibitory behaviour of FI-CR1-DAF is due to the indirect actions of C3-convertase and C5-convertase (Fig. 1). Trajectories labelled with “ *ζ*” represent production of FI-CR1-DAF.

The system inducts MAC along with PICyts to clear the pathogen though lysis and inflammation, respectively (Chouaki Benmansour, Carvelli & Vivier, 2021). Transitions labelled with “ *ω*” indicate inhibition of CoV2 due to co-existence of MAC and PICyts. The entities C3 and PICyts are indirectly involved in the production of MAC that are being represented by the transitions labelled with “ *ϕ*” in Fig. 5. Separately, PICyts can also induce MAC that is shown in transitions labelled with “ *ρ*” .

In normal condition, most probable biological periodic cycles termed as homeostatic behaviour were found which includes SCC-1 and SCC-2 (Fig. 5). The cyclic behaviour between state $(0,0,1,0,0,0)$ and state $(0,0,1,0,0,1)$ is represented by SCC-1. The state $(0,0,1,0,0,0)$ has prominent BC among the other states of SCC-1. Between these two states, the cyclic trajectory indicates recurrent induction of PICyts. Trajectory $(0,1,0,0,0,1)$ $\to$ $(0,1,1,0,0,1)$ $\to$ $(0,0,1,0,0,1)$ $\to$ $(0,0,1,0,0,0)$ represents immunosuppression due to the presence of FI-CR1-DAF. SCC-2 shows significance of the cyclic trajectory initial state $(0,1,0,1,1,1)$ and terminal state $(0,1,0,2,1,1)$ . Between these two states the cyclic path represents recurrent induction of PICyts. Immunosuppression due to active FI-CR1-DAF is represented by trajectories $(0,1,0,0,1,1)$ $\to$ $(0,1,1,0,1,1)$ $\to$ $(0,1,1,0,1,0)$ $\to$ $(0,0,1,0,1,0)$ , the states in the trajectories have maximum BC as compared to other states. In the SCCs, the entities CoV2, C3, C5a, and PICyts performed oscillatory behaviours except CoV2 and MAC. In SCC-1, CoV2 and MAC continuously remained inactive. While in SCC-2, CoV2 constantly remained inactive and MAC active.

In summary, during normal signalling the entities fluctuates in irregular fashion. The system leading toward hyper-inflammatory state $(0,1,1,2,1,1)$ . After completing successful immune response, all the trajectories eventually converge to an attractor as stable normal state $(0,0,0,0,0,0)$ .

### Signalling in pathogenic condition

Modelling of the pathogenic condition was conducted by utilizing logical parameters set tabulated in Table 3, that represents Model-20 (M-20) is retrieved from logical parameters sets (models) are provided in File S7. The models have also been shown *via* heatmap (Fig. S5). Most of the logical parameters for pathogenic condition are same as logical parameters of entities taken for normal signalling (Table 2) with some exceptions that is ${K_{CoV2}}\{ MAC,PICyts\} = 1$ , ${K_{C3}}\{ PICyts,FI - CR1 - DAF\} = 1$ , ${K_{C5a}}\{ PICyts,FI - CR1 - DAF\} = 1$ and ${K_{C5a}}\{ PICyts\} = 1$ are different. ${K_{CoV2}}\{ MAC,PICyts\} = 1$ represent recurrent infection. For this study basically, intervened in CTL given in Eq. (1). With the eliminated sub-formula ${\Psi _\beta }$ as second characteristics, we have $\Psi = {\Psi _\alpha } \wedge {\Psi _\gamma }$ as CTL.

**Table 3:** The logical parameters set for modelling pathogenic condition signalling. The logical parameters set represents Model-20 is retrieved from parameters sets are provided in File S7. The BRN (GINSim, File S8) for pathogenic condition signalling is parameterized by using these parameters.

S. No.	Logical parameters
1	${K_{CoV2}}\{ \} = 0$
2	${K_{CoV2}}\{ MAC\}$ = 0
3	${K_{CoV2}}\{ PICyts\}$ = 0
4	${K_{CoV2}}\{ MAC,PICyts\}$ = 1
5	${K_{C3}}\{ \}$ = 0
6	${K_{C3}}\{ CoV2\}$ = 1
7	${K_{C3}}\{ FI - CR1 - DAF\}$ = 0
8	${K_{C3}}\{ PICyts\}$ = 1
9	${K_{C3}}\{ CoV2,FI - CR1 - DAF\}$ = 1
10	${K_{C3}}\{ FI - CR1 - DAF,PICyts\}$ = 1
11	${K_{C3}}\{ CoV2,PICyts\}$ = 1
12	${K_{C3}}\{ CoV2,FI - CR1 - DAF,PICyts\}$ = 1
13	${K_{C5a}}\{ \}$ = 0
14	${K_{C5a}}\{ C3\}$ = 1
15	${K_{C5a}}\{ FI - CR1 - DAF\}$ = 0
16	${K_{C5a}}\{ PICyts\}$ = 1
17	${K_{C5a}}\{ C3,FI - CR1 - DAF\}$ = 1
18	${K_{C5a}}\{ FI - CR1 - DAF,PICyts\}$ = 1
19	${K_{C5a}}\{ C3,PICyts\}$ = 1
20	${K_{C5a}}\{ C3,FI - CR1 - DAF,PICyts\}$ = 1
21	${K_{MAC}}\{ \}$ = 0
22	${K_{MAC}}\{ C3\}$ = 1
23	${K_{MAC}}\{ PICyts\}$ = 1
24	${K_{MAC}}\{ C3,PICyts\}$ = 1
25	${K_{FI - CR1 - DAF}}\{ \}$ = 0
26	${K_{FI - CR1 - DAF}}\{ PICyts\}$ = 1
27	${K_{PICyts}}\{ \}$ = 0
28	${K_{PICyts}}\{ C3\}$ = 0
29	${K_{PICyts}}\{ C5a\}$ = 2
30	${K_{PICyts}}\{ FI - CR1 - DAF\}$ = 0
31	${K_{PICyts}}\{ C3,C5a\}$ = 2
32	${K_{PICyts}}\{ C5a,FI - CR1 - DAF\}$ = 2
33	${K_{PICyts}}\{ C3,FI - CR1 - DAF\}$ = 1
34	${K_{PICyts}}\{ C3,C5a,FI - CR1 - DAF\}$ = 2

The BRN shown in Fig. 3 (GINSim, File S8) that is simulated to state graph (GINSim File S9) is visualized *via* graphviz (Fig. S6). The pathogenic condition signalling of different entities depicted in Fig. 6 is further used for interpretation.

The persistent existence of infection (recurrent infection) can cause harsh immune response (Paracha et al., 2014). Previous experimental studies have been admitted SARS-CoV-2 reinfection or mutation of the original virus in the host’s body (Zapor, 2020; Ren et al., 2022). In this study, continuous presence of the infection was the main difference between normal and pathogenic signalling.

As compared to normal signalling, new event of continuous presence of infection or regeneration of CoV2 was found represented by trajectories labelled with “ *ε*” . The trajectories include $(0,0,0,0,0,0)$ $\to$ $(1,0,0,0,0,0)$ , $(0,0,1,0,0,0)$ $\to$ $(1,0,1,0,0,0)$ and $(0,0,1,0,0,1)$ $\to$ $(1,0,1,0,0,1)$ . Moreover, during pathogenic signalling normal state $(0,0,0,0,0,0)$ was found unstable (Fig. 6B).

The initial phase signalling was comparable to normal signalling in which homeostatic responses were observed indicated by SCC-1 to SCC-4 in which most of the states were at immunosuppression. Degradation of PICyts level due to FI-CR1-DAF indicated by trajectories labelled with “ *β*” and FI-CR1-DAF mediated downregulation of C5a and C3 represented by trajectories labelled with “ *ϕ*” and “ *λ*” plays pivotal role in immunosuppression, whereas in late phase signalling re-induction of CoV2 and deadlock state $(0,1,1,2,1,1)$ were found as main feature of pathogenic condition shown in Fig. 6B.

The main state transitions that enhanced the expression level of PICyts and lead towards deadlock state $(0,1,1,2,1,1)$ in late phase includes activation of C5a due to active C3 indicated by trajectories labelled with “ *δ*” and activation of C5a due to loss of FI-CR1-DAF represented by trajectories labelled with “ *Δδ*” .

Trajectories labelled with “ *ρ*” and “ *γ*” denoting PICyts and C3 mediated upregulation of MAC. Compare to normal signalling at which MAC and PICyts co-exist to repress the virions, here we additionally found individual influence of MAC and PICyts in repression of the virions indicated by state transitions labelled with “ *Δθ*” and “ *θ*” , respectively.

In summary, overall pattern of pathogenic signalling implied oscillatory behaviours of entities particularly PICyts observed in the SCCs in early phase of signalling. Deviation of state transitions from the SCCs in late phase signalling strengthen PICyts expression level leading to an attractor as hyper-inflammatory stable state $(0,1,1,2,1,1)$ and re-induction of CoV2.

### Intervention modelling

In literature, studies performed on intervention for modelling of sepsis, a strategy like removing and changing of entities interaction followed (Paracha et al., 2014). In the study of cancer, intervention performed by elimination of the CTL property (Saeed et al., 2018).

Basically, intervention is possible due to mutation and/or therapeutic intervention that can alter the role of resources and state graph behaviour/s (Paracha et al., 2014). It is helpful to suggest potential therapeutic target. Though intervention, deadlock state can be removed, and the biological system leads towards normal homeostatic behaviours termed as SCCs. The interventions were utilized to analyze altered signalling events that effects on states stability and periodic cycles. Moreover, comparative assessment with intact (non-intervened) signalling.

The intervention modelling of the BRN signalling dynamics have been discussed in case-1 and case-2, respectively. For both cases, the logical parameters were retrieved *via* formal verification model checker. In case-1, changed logical parameters values such as ${K_{C5a}}\{ C3\}$ and ${K_{C5a}}\{ C3,PICyts\}$ values from 1 to 0 in “PARAMETER” section for input of model checker. In case-2, the CTL for pathogenic condition was intervened that is altered C5a value from 1 to 0 in sub-formula “ Ψα” .

#### Case-1 (Intervention in C3 mediated upregulation of C5a and PICyts)

Intervention in C3 mediated upregulation of C5a and PICyts during pathogenic condition is considered. An activator C3 of C5a and PICyts has been targeted in different experimental studies (Ram Kumar Pandian et al., 2020; de Nooijer et al., 2021). For this study, the logical parameters set tabulated in Table 4 indicates M-8 retrieved from parameters sets are given as File S10, that are also been represented through heatmap (Fig. S7). Most of the logical parameters are same as pathogenic condition parameters set (Table 3) except some changed logical parameters such as ${K_{C5a}}\{ C3\} = 0$ , ${K_{C5a}}\{ PICyts\}$ = 0, ${K_{C5a}}\{ FI - CR1 - DAF,PICyts\} = 0$ , ${K_{C5a}}\{ C3,PICyts\}$ = 0 and ${K_{PICyts}}\{ C3,FI - CR1 - DAF\}$ = 0.

**Table 4:** The logical parameters sets for modelling intervention (INT) cases-1 and case-2. The logical parameters sets used in modelling of the BRNs (GINSim; Files S11 and S14) for intervened signalling. For both cases, interventions in CTL formula of pathogenic condition have discussed in result section. INT case-1 indicates parameters set (Model-8) selected from parameters sets are given in File S10. INT case-2 indicates Model-18 selected from parameters sets are given in File S13.

S. No.	Logical parameters	INT case-1	INT case-2
1	${K_{CoV2}}\{ \}$	0	0
2	${K_{CoV2}}\{ MAC\}$	0	0
3	${K_{CoV2}}\{ PICyts\}$	0	0
4	${K_{CoV2}}\{ MAC,PICyts\}$	1	1
5	${K_{C3}}\{ \}$	0	0
6	${K_{C3}}\{ CoV2\}$	1	1
7	${K_{C3}}\{ FI - CR1 - DAF\}$	0	0
8	${K_{C3}}\{ PICyts\}$	1	1
9	${K_{C3}}\{ CoV2,FI - CR1 - DAF\}$	1	1
10	${K_{C3}}\{ FI - CR1 - DAF,PICyts\}$	1	1
11	${K_{C3}}\{ CoV2,PICyts\}$	1	1
12	${K_{C3}}\{ CoV2,FI - CR1 - DAF,PICyts\}$	1	1
13	${K_{C5a}}\{ \}$	0	0
14	${K_{C5a}}\{ C3\}$	0	1
15	${K_{C5a}}\{ FI - CR1 - DAF\}$	0	0
16	${K_{C5a}}\{ PICyts\}$	0	1
17	${K_{C5a}}\{ C3,FI - CR1 - DAF\}$	1	1
18	${K_{C5a}}\{ FI - CR1 - DAF,PICyts\}$	0	1
19	${K_{C5a}}\{ C3,PICyts\}$	0	1
20	${K_{C5a}}\{ C3,FI - CR1 - DAF,PICyts\}$	1	1
21	${K_{MAC}}\{ \}$	0	0
22	${K_{MAC}}\{ C3\}$	1	1
23	${K_{MAC}}\{ PICyts\}$	1	1
24	${K_{MAC}}\{ C3,PICyts\}$	1	1
25	${K_{FI - CR1 - DAF}}\{ \}$	0	0
26	${K_{FI - CR1 - DAF}}\{ PICyts\}$	1	1
27	${K_{PICyts}}\{ \}$	0	0
28	${K_{PICyts}}\{ C3\}$	0	0
29	${K_{PICyts}}\{ C5a\}$	2	0
30	${K_{PICyts}}\{ FI - CR1 - DAF\}$	0	0
31	${K_{PICyts}}\{ C3,C5a\}$	2	0
32	${K_{PICyts}}\{ C5a,FI - CR1 - DAF\}$	2	2
33	${K_{PICyts}}\{ C3,FI - CR1 - DAF\}$	0	1
34	${K_{PICyts}}\{ C3,C5a,FI - CR1 - DAF\}$	2	2

Modelling of the BRN given in Fig. 3 (GINSim File S11) was conducted by utilizing logical parameters set (Table 4). The state graph (GINSim File S12) visualized *via* graphviz is given in Fig. S8. The state graph shown in Fig. 7 is further used for interpretation.

The configured logical parameters set form basis to remove deadlock state and restore homeostasis. The BRN produced state graph that does not have deadlock state $(0,1,1,2,1,1)$ . Trajectories lead to normal immune response $(0,0,0,0,0,0)$ and homeostatic behaviours indicated by SCC-1 to SCC-4 in Fig. 7B.

In general, the state graph state transitions signalling patterns comparable with normal signalling as shown in Fig. 5, but some trajectories were found different as recurrent induction of the infection (paths labelled with “ *ε*” ). Trajectory labelled with “ *Δμ*” representing induction of C3 due to simultaneous presence of CoV2 and PICyts. Trajectories labelled with “ *Δη*” represent loss of FI-CR1-DAF with presence/absence of CoV2 and presence of PICyts mediated upregulation of C3. Trajectories labelled with “ *Δϕ*” indicate loss of FI-CR1-DAF induction of C3 due to presence of CoV2. C3 mediated production of MAC are represented by trajectories labeled with “ *γ*” and the MAC generated further repressed CoV2 indicated by trajectories labelled with “ *Δθ*” . Moreover, PICyts produced by C3 and C5a that degraded CoV2 represented by trajectories labelled with “ *θ*” . In experimental studies, it is known that activated PICyts due to complement upregulating factors caused inflammatory response against the pathogen. Comparatively in normal condition, both MAC and PICyts co-exist for inhibition of CoV2 represented by trajectories labelled with “ *ω*” shown in Fig. 5B.

On the whole most state transitions were found leading toward normal immune response $(0,0,0,0,0,0)$ in the absence of active CoV2. These circumstances were true excluding some prominent hyper-inflammatory response states with co-existence of C3 and C5a. The states include $(1,1,1,2,1,1)$ , $(1,1,1,2,1,0)$ , $(1,1,1,2,0,1)$ , $(0,1,1,2,0,0)$ , and $(0,1,1,2,1,0)$ labelled with an asterisk (*) shown in Fig. 7B. These states were identified with high chance to generate overactive immune response $(0,1,1,2,1,1)$ was found unstable. Overall BRN signalling dynamics (Fig. 7B) were found similar to pathogenic BRN signalling dynamics (Fig. 6B) with exceptional difference found as absence of an attractor deadlock state $(0,1,1,2,1,1)$ in the state graph of intervened case. Homeostasis was observed in all phases of signalling whereas in pathogenic condition homeostasis found only in the early phase of signalling.

It is of note that the intervened signalling yield homeostasis with sustain oscillations of entities in the SCCs were observed. The qualitative model performed asymptotic behaviour that is state transitions reached to hyper-inflammatory state $(0,1,1,2,1,1)$ then declined to recovery state $(0,0,0,0,0,0)$ in late phase and the system remained in periodic cycles. The study implied C3 a necessary target.

#### Case-2 (Intervention in C5a mediated upregulation of PICyts)

Intervention in C5a mediated upregulation of PICyts is considered in this intervention study. In SARS-CoV-2 infection high level of C5a increased concentration levels of PICyts and caused cytokine storm (Ram Kumar Pandian et al., 2020). In the BRN (Fig. 3) a positive loop between C5a and PICyts exist, direct correlation of SARS-CoV2 infection severity with C5a and provoke of PICyts by C5a revealed C5a as necessary target, that has been targeted in many experimental studies (Ram Kumar Pandian et al., 2020; Carvelli et al., 2020; de Nooijer et al., 2021). Compare to pathogenic condition logical parameters set (Table 3) the only changed logical parameters are ${K_{PICyts}}\{ C5a\} = 0$ and ${K_{PICyts}}\{ C3,C5a\}$ = 0. The logical parameters set tabulated in Table 4 represents M-18 selected from logical parameters sets are given in File S13. The logical parameters sets have also been presented through heatmap (Fig. S9).

The parameters set (Table 4) was used for modelling the BRN is shown in Fig. 3 (GINSim; File S14) is simulated to state graph (GINSim; File S15) that is visualized through graphviz in Fig. S10. The state graph shown in Fig. 8 is further used for interpretation.

Overall qualitative model signalling patterns were comparable with case-1 and slight difference. Trajectories labelled with “ *η*” indicate PICyts mediated induction of C3 with presence/absence of CoV2. Trajectories labelled with “ *Δδ*” indicating loss of FI-CR1-DAF mediated upregulation of C5a with the presence of C3. Among important states, $(0,1,1,0,1,0)$ and $(0,0,1,0,1,0)$ were identified less important relatively the same states present in case-1 having high betweenness centrality.

Compared to normal and pathogenic conditions state graph attractors stable normal state $(0,0,0,0,0,0)$ and deadlock state $(0,1,1,2,1,1)$ were found absent. While recurrent infection represented by trajectory $(0,0,0,0,0,0)$ $\to$ $(1,0,0,0,0,0)$ labelled with “ *ε*” in the SCCs such as SCC-1 and SCC-3 are shown in Fig. 8B was comparable with pathogenic condition.

It is of note that inflammatory response due to concentration levels of PICyts were observed in regular fashion in all SCCs for lowering titre of SARS-CoV-2 throughout entire phases of signalling and maintained homeostatic response. While the inflammatory responses were found present in pathogenic condition state graph SCCs only in early phase signalling but in late phase of signalling hyper-inflammation responses observed. In pathogenic signalling, C3 and C5a mediated induction of PICyts level remained high in the terminal states. The transition of states tend to hyper-inflammatory stable state $(0,1,1,2,1,1)$ .

However, this intervention study inferred that C5a as up regulator of PICyts was identified an important target. Suppression of PICyts levels through this intervention mechanism diverted hyper-inflammatory state transitions to normal homeostatic behaviours during SARS-CoV-2 infection.

## Discussion

The complement system (CS) is an essential part of innate immunity that provides protection against invading pathogens by producing anaphylatoxins, opsonin’s, and membrane attack complexes, *etc*. (Zewde et al., 2021; Zewde & Morikis, 2018). Like other infections, SARS-CoV-2 can activate CS as well. However, the abnormal dysregulation of CS leads to the overactivation of the system that is primarily associated with the pathogenesis of SARS-CoV-2 infection (Yu et al., 2022; Sinkovits et al., 2021). In this overactivated response, cytokines are produced that are not only inducing the immune response against SARS-CoV-2 but also causing multiple organ damages (Chouaki Benmansour, Carvelli & Vivier, 2021). Understanding the general dysregulation and over-activation processes of the CS is crucial for tailoring the beneficial responses by intervening the damages to the host.

Systems biology can provide useful information regarding human health and diseases by modelling the complex behaviours of the system (Banerjee, Chakraborty & Ray, 2022). Several studies have been performed by using systems biology approaches to understand the complex behaviours of the human immune systems successfully by employing kinetic logic (Paracha et al., 2014) and Boolean logic approaches (Saeed et al., 2016, 2018; Aslam et al., 2014). For the complement pathways, the interaction of SARS-CoV-2 with human genes was modelled by *in silico* techniques. The model predictions were validated *via* the KEGG pathway in protein set enrichment analysis of transcriptomics and proteomics data sets reported previously for the virus-infected Huh7 cell-lines (Tiwari et al., 2020). We try to establish the first qualitative model BRN for the SARS-CoV-2 induced complement cascades, that modelled the signalling pattern leads to overactivation response and its management *via* intervention. The model results were found promising with the experimental studies reported. Researchers are highly interested *in vivo* and *in silico* studies for the downregulation of CS activity as a therapeutic agent to suppress the inflammatory response (Ostrycharz & Hukowska-Szematowicz, 2022), it could be modelled in our case. Intervened qualitative model for the management of hyper-inflammation could be extended to explore the suppression of hyper-inflammation response. For this the signalling pattern for the inhibitors-mediated downregulation of PICyts could be modelled. In this study, activators-mediated upregulation of PIyts was addressed to manage the hyper-inflammation response.

SARS-CoV-2 infection stimulates innate immunity that produces pro-inflammatory cytokines (PICyts) (Diamond & Kanneganti, 2022). The acute phase of inflammation tries to fight the virus during the infection, but hyper-activation of innate immunity generated cytokine storm leading to chronic inflammation results in multiple organ failure and autoimmune disorders (Vinciguerra et al., 2020; Wang et al., 2022). The activation of CS is found in the pathophysiology of acute respiratory distress syndrome (ARDS) with increased levels of plasma complement-positive regulators, particularly higher expression levels of complement anaphylatoxins C5a and C3a that are the main mediators of PICyts (Vinciguerra et al., 2020). The formation of MAC can suppress the virus through the lysis mechanism (Chouaki Benmansour, Carvelli & Vivier, 2021).

In this study, SARS-CoV-2 induced CS regularity pathways were developed based on previous experimental findings related with the entities of complement system (Fig. 1). The pathways were reduced (Table 1) to construct the BRN (Fig. 3). The BRN was parameterized with various sets of parameters and simulated to produce state graphs for analysis of different signalling patterns. Normal, pathogenic and intervened models were considered in this study. Network analysis of state graphs were performed based on betweenness centrality analysis for the selection of important states, transitions and cyclic (SCCs).

Sequential expression levels of entities were observed in the cycles. The successive oscillatory behaviours between up-and-down regulation of PICyts were found. In experimental studies, the swing in PICyts expression levels have been reported (García, 2020). Inflammatory response leads to hyper-inflammatory response and eventually reduction in inflammation as suppressed immune response has been observed during SARS-CoV-2 infection. In this study, hyper-inflammation can be associated with the states in which PICyts at elevated level “ 2” . Inflammation can be associated with PICyts at level “ 1” . The decrease in immune response can be associated with low level of PICyts “ 0” . The decrease in expression level of PICyts due to the presence of FI-CR1-DAF in the system was observed. The severity of the infection was shown by the states indicated with an asterisk (*) in the state graphs where C3 and C5a were active and PICyts was hyperactive simultaneously. States with a high likelihood of stabilising an excessive immune response tend to affect the system. PICyts and/or MAC mediated lowering of CoV2 titre was found interesting that could be helpful in mitigation and control of the infection severity.

Trajectories of normal and pathogenic conditions were found comparable during the initial phase. The distinct signalling patterns were observed in late phase. Normal signalling leads to an attractor $(0,0,0,0,0,0)$ indicating the successful immune response. This could be due to the active FI-CR1-DAF in the late phase and induction of MAC and/or PICyts throughout the system signalling pattern. However, pathogenic signalling tends to lead the system to $(0,1,1,2,1,1)$ representing overactive immune response.

Moreover, an important attribute of the pathogenic model is the absence of a stable normal state and recurrent infection as continuous presence of SARS-CoV-2 depicted by state transition like $(0,0,0,0,0,0)$ $\to$ $(1,0,0,0,0,0)$ . The recurrent infection is due to inactive PICyts and/or MAC result in increased CoV2 load. This may represent that lack of inflammation response due to low expression levels of PICyts and insufficient lysis of the virus due to unavailability of MAC during SARS-CoV-2 infection may be a cause of recurrent infection (Garg et al., 2021; Abu-Raddad, Chemaitelly & Bertollini, 2021). Moreover, due to fluctuation in the levels of FI-CR1-DAF, the levels of PICyts also fluctuated as a result of increase in CoV2 load.

Intervention modelling for the management of hyper-inflammation was performed. We explored some significant results, in which the entities remained in oscillatory behaviours. The system diverted overactive immune response to homeostatic responses. Interestingly, in intervened signalling of C3 mediated upregulation of C5a and PICyts, we found some trajectories represented by “ *Δα*” in which active FI-CR1-DAF incapable to inhibit PICyts. Such behaviour is not present in intervened signalling of C5a mediated upregulation of PICyts but available in normal and pathogenic signalling.

In many states activated C3 upregulates C5a and PICyts simultaneously or separately. In some states, FI-CR1-DAF more effectively suppresses C3, C5a, and PICyts. It has been shown that during some state transitions, the immune system is temporarily unable to clear the viral load, which suggests an impaired immunological response. In some states during pathogenic signalling, loss of FI-CR1-DAF can be harmful and the production of C3, C5a, and PICyts alter the behaviour of immune response. Excessive innate immune response and detrimental high levels of PICyt results in the form of a cytokine storm that have been reported during SARS-CoV-2 infection.

**Figure 9:**
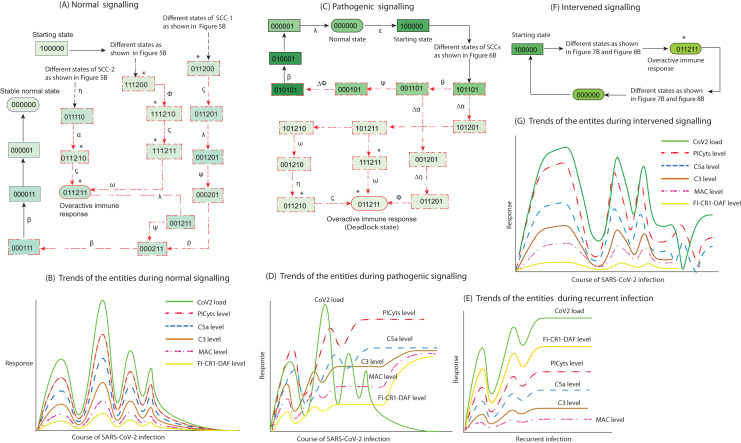
Inference in this study. The entities interaction different signalling events were observed in this study, that are depicted in less involved Figures A, C, and F. (See the state transitions signalling labelled with small Greek letters in Figs. 5–8). The state transitions can be used to draw hypothetical trends of the entities response shown for normal signalling (B), pathogenic signalling (D and E), and intervened signalling (G). The signalling events can effect on CoV2 load, induction of PICyts, and it mediators C5a and C3. Production of MAC because of PICyts and C3 induction. FI-CR1-DAF mediated suppression of PICyts, C5a, and C3. In normal signalling, the behaviour of FI-CR1-DAF limited the discrete values of PICyts, C5a, and C3. The PICyts and MAC generation can mitigate the CoV2 load and the system ultimately tends toward normal level. In pathogenic signalling, the levels of entities tend to overactive immune response as hyper-inflammatory stable state. During recurrent signalling (E), it is proposed that the increase level of FI-CR1-DAF inhibits PICyts, C5a, and C3. The suppressed levels of PICyts and C3 are inadequate to increase MAC level. Low level of MAC formation and PICyts production unable to inhibit CoV2 and the titre of CoV2 is increased. In intervened signalling, the levels of entities are controlled. The entities performed homeostatic response with sustain pattern of oscillations as shown in (G).

In summary, during normal signalling pattern the FI-CR1-DAF restricted the levels of PICyts. Moreover, MAC and/or PICyts were able to reduce the virus load successfully and the system leads to recovery immune response. On the other hand, during pathogenic signalling, the decrease of the virus load was found not beneficial. The PICyts remain at elevated levels and the system leads to over activated immune response and recurrent infection. The management of PICyts was successful *via* intervention and the system diverted to the homeostatic response. The normal, pathogenic, and intervened signalling of entities in state graphs shown in Figs. 5–8 are summarized in Fig. 9.

## Conclusions

In conclusion, the qualitative modelling of the complement system signalling pathways suggested that the immune system was overreacting in a way that is managed differently in different scenarios. Signalling may produce various outcomes if the complement entities lose a certain pattern of up-and-down regulation of proteins. In this study, an effort is made to highlight the patterns of the entities necessary to keep a balance in a successful immune response after inflammation by using the kinetic logic approach. The results implied the pros and cons of PICyts and MAC responses. The results indicated that a characteristic of the pathogenic infection situation is the persistent elevation of SARS-CoV-2 titre. It was shown in infection normal condition signalling that homeostatic behaviours from an overly active response trend toward an effective immune response. This trait suggests that in COVID-19 individuals, a hyper-inflammatory response is followed by immunomodulation and a reduction in inflammation. Homeostatic responses are brought about by the pattern of overexpression and subsequent downregulation of PICyts, which, also changes how C3 and C5a are activated. For the management of hyper-inflammation, an intervention modelling is also being performed. The results inferred that the PICyts level can be controlled and mitigated through intervention in C3 and C5a activation levels. It has been suggested that targeting C3 and C5a may provide better therapeutic control strategy for the treatment of SARS-CoV-2 infection. However, this study provide a foundation and a course for subsequent translational research with experimental verification.

## Supplemental Information

10.7717/peerj.15794/supp-1 Supplemental Information 1 Examples of dummy BRNs.

10.7717/peerj.15794/supp-2 Supplemental Information 2 Normal condition parameters set heatmap.

10.7717/peerj.15794/supp-3 Supplemental Information 3 Normal condition parameters set dummy tentency graphs.

10.7717/peerj.15794/supp-4 Supplemental Information 4 Normal condition state graph.

10.7717/peerj.15794/supp-5 Supplemental Information 5 Pathogenic condition parameters sets heatmap.

10.7717/peerj.15794/supp-6 Supplemental Information 6 Pathogenic condition state graph.

10.7717/peerj.15794/supp-7 Supplemental Information 7 Intervention Case 1 parameters sets heatmap.

10.7717/peerj.15794/supp-8 Supplemental Information 8 Intervention Case 1 state graph.

10.7717/peerj.15794/supp-9 Supplemental Information 9 Intervention Case 2 parameters sets heatmap.

10.7717/peerj.15794/supp-10 Supplemental Information 10 Intervention Case 2 state graph.

10.7717/peerj.15794/supp-11 Supplemental Information 11 Definitions.

10.7717/peerj.15794/supp-12 Supplemental Information 12 CTL Fundamentals.

10.7717/peerj.15794/supp-13 Supplemental Information 13 Normal condition parameters sets.

10.7717/peerj.15794/supp-14 Supplemental Information 14 Logical Parameters Evidences.

10.7717/peerj.15794/supp-15 Supplemental Information 15 Normal BRN.

10.7717/peerj.15794/supp-16 Supplemental Information 16 Normal state graph model code.

10.7717/peerj.15794/supp-17 Supplemental Information 17 Pathogenic condition parameters sets.

10.7717/peerj.15794/supp-18 Supplemental Information 18 Pathogenic BRN.

10.7717/peerj.15794/supp-19 Supplemental Information 19 Pathogenic state graph.

10.7717/peerj.15794/supp-20 Supplemental Information 20 Intervention Case 1.

10.7717/peerj.15794/supp-21 Supplemental Information 21 Intervention Case 1 BRN.

10.7717/peerj.15794/supp-22 Supplemental Information 22 Intervention Case 2 state graph.

10.7717/peerj.15794/supp-23 Supplemental Information 23 Intervention Case 2.

10.7717/peerj.15794/supp-24 Supplemental Information 24 Intervention Case 2 BRN.

10.7717/peerj.15794/supp-25 Supplemental Information 25 SMBioNet and GINSim kinetic parameters entry.

10.7717/peerj.15794/supp-26 Supplemental Information 26 Dummy model parameters set.

10.7717/peerj.15794/supp-27 Supplemental Information 27 Experimental observations.

10.7717/peerj.15794/supp-28 Supplemental Information 28 Normal condition state graph rendered in cytoscape (CYS).

10.7717/peerj.15794/supp-29 Supplemental Information 29 Normal condition state graph rendered in cytoscape (SIF).

10.7717/peerj.15794/supp-30 Supplemental Information 30 Pathogenic condition state graph rendered in cytoscape (CYS).

10.7717/peerj.15794/supp-31 Supplemental Information 31 Pathogenic condition state graph rendered in cytoscape (SIF).

10.7717/peerj.15794/supp-32 Supplemental Information 32 Intervention Case 1 state graph rendered in cytoscape (CYS).

10.7717/peerj.15794/supp-33 Supplemental Information 33 Intervention Case 1 state graph rendered in cytoscape (SIF).

10.7717/peerj.15794/supp-34 Supplemental Information 34 Intervention Case 2 state graph rendered in cytoscape (CYS).

10.7717/peerj.15794/supp-35 Supplemental Information 35 Intervention Case 2 state graph rendered in cytoscape (SIF).

## Additional Information and Declarations

### Data Availability

The following information was supplied regarding data availability: The model codes, algorithm of model checker in the SMBioNet tool code along with logical parameters sets and the BRN simulated to state graphs rendered in cytoscape are available in the Supplemental Files.
