# Supplementary figures and images for: Modelling and analysis of the complement system signalling pathways: roles of C3, C5a and pro-inflammatory cytokines in SARS-CoV-2 infection
Source: PeerJ. 2023 Sep 20;11:e15794. doi: 10.7717/peerj.15794 (PMC10517668; doi:10.7717/peerj.15794)

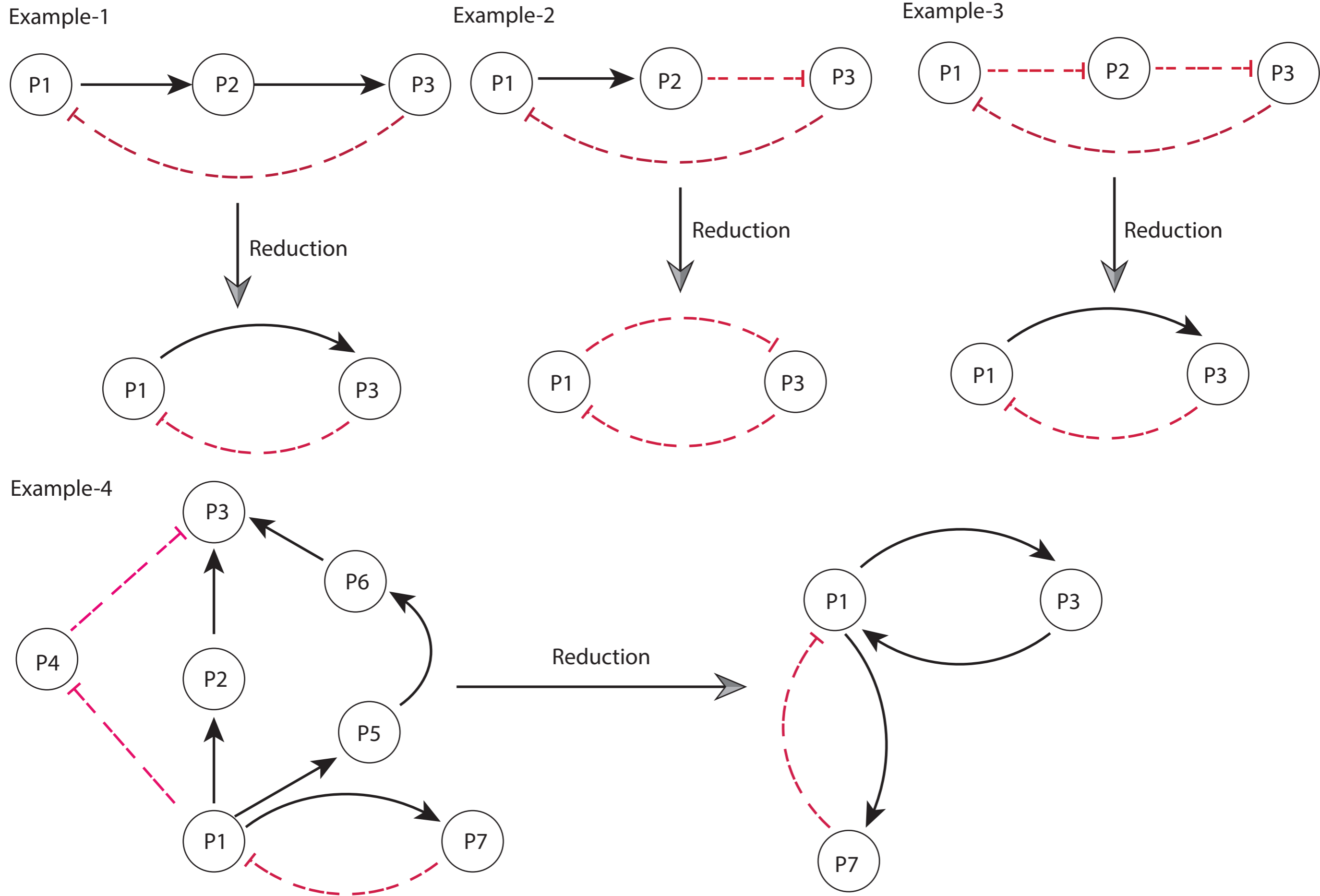

Supplement: Supplemental Information 1 [file peerj-11-15794-s001.pdf]

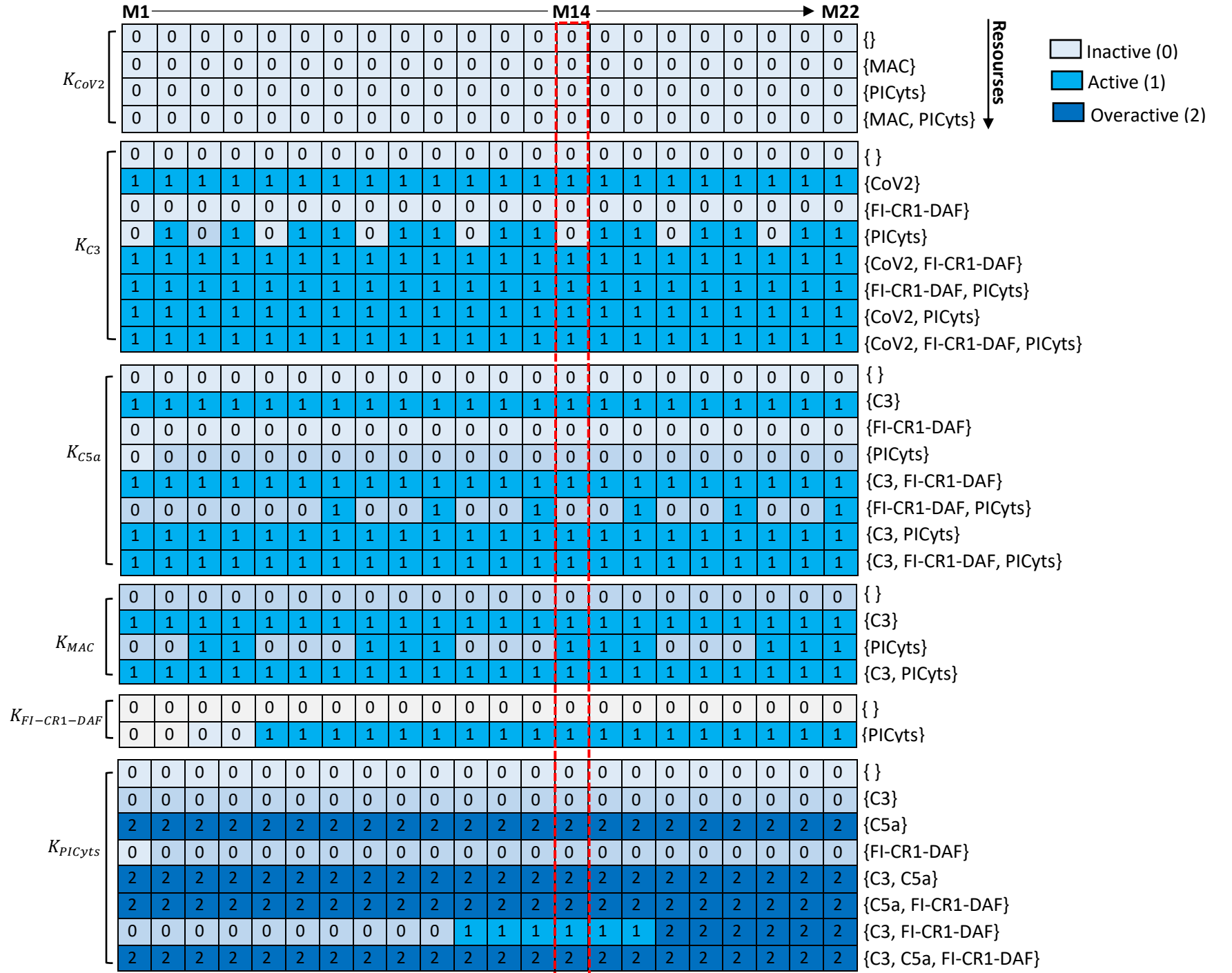

**Figure:** For normal condition heatmap representation of logical parameters sets.

Supplement: Supplemental Information 2 [file peerj-11-15794-s002.pdf]
