# Supplementary material for: Modelling and analysis of the complement system signalling pathways: roles of C3, C5a and pro-inflammatory cytokines in SARS-CoV-2 infection
Source: PeerJ. 2023 Sep 20;11:e15794. doi: 10.7717/peerj.15794 (PMC10517668; doi:10.7717/peerj.15794)
Supplement: Supplemental Information 3 [file peerj-11-15794-s003.pdf]

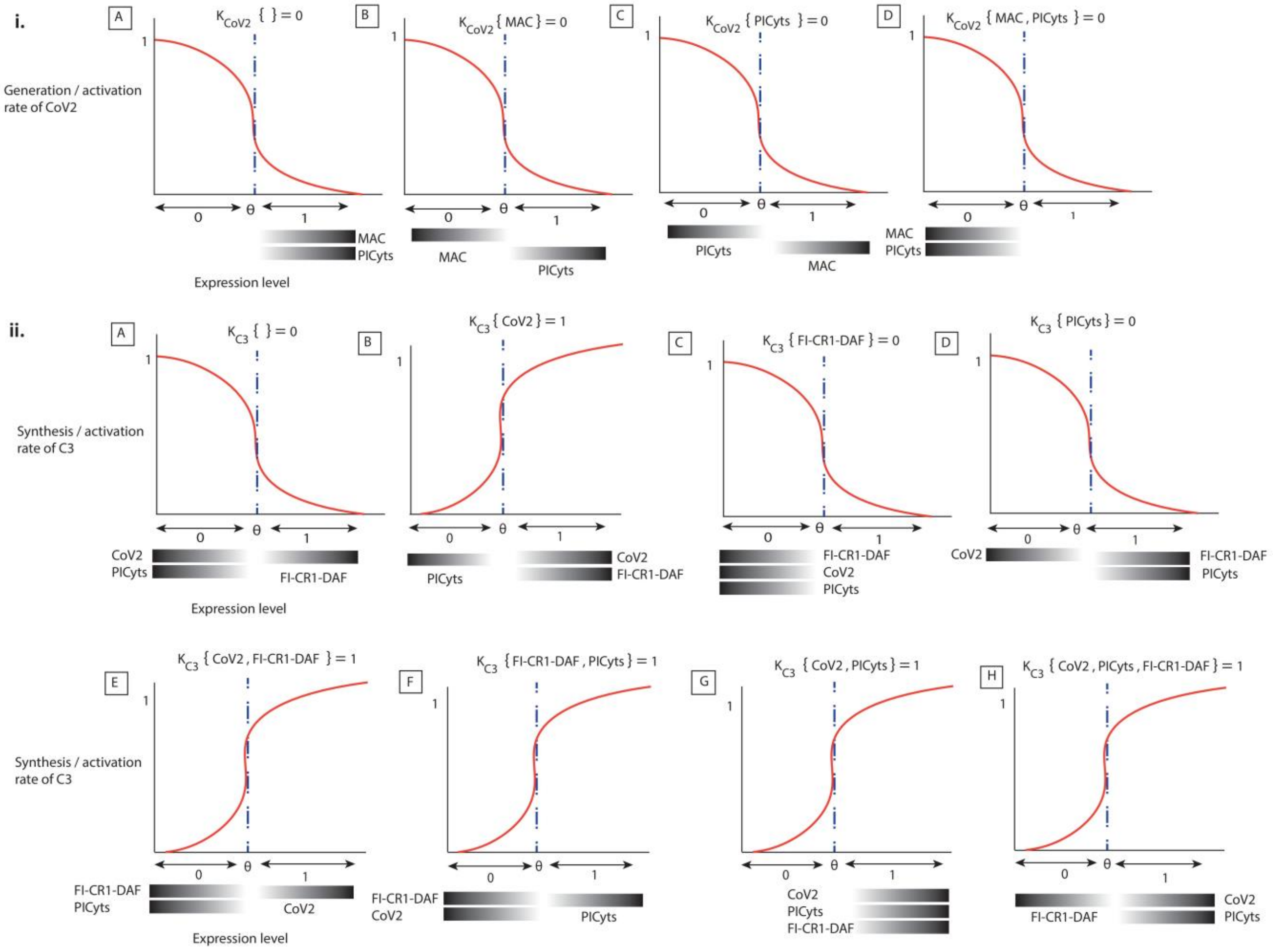

Figure. Dummy tendency graphs. For normal condition, the logical parameters given in Table 2 have been shown through dummy tendency graphs. Along x-axes resource entities and y-axes generation/synthesis level of targeted entities are taken. Logically absence/presence of negative regulator/s as resource indicating it is actually active/inactive for downregulation of the targeted entities [1,2].

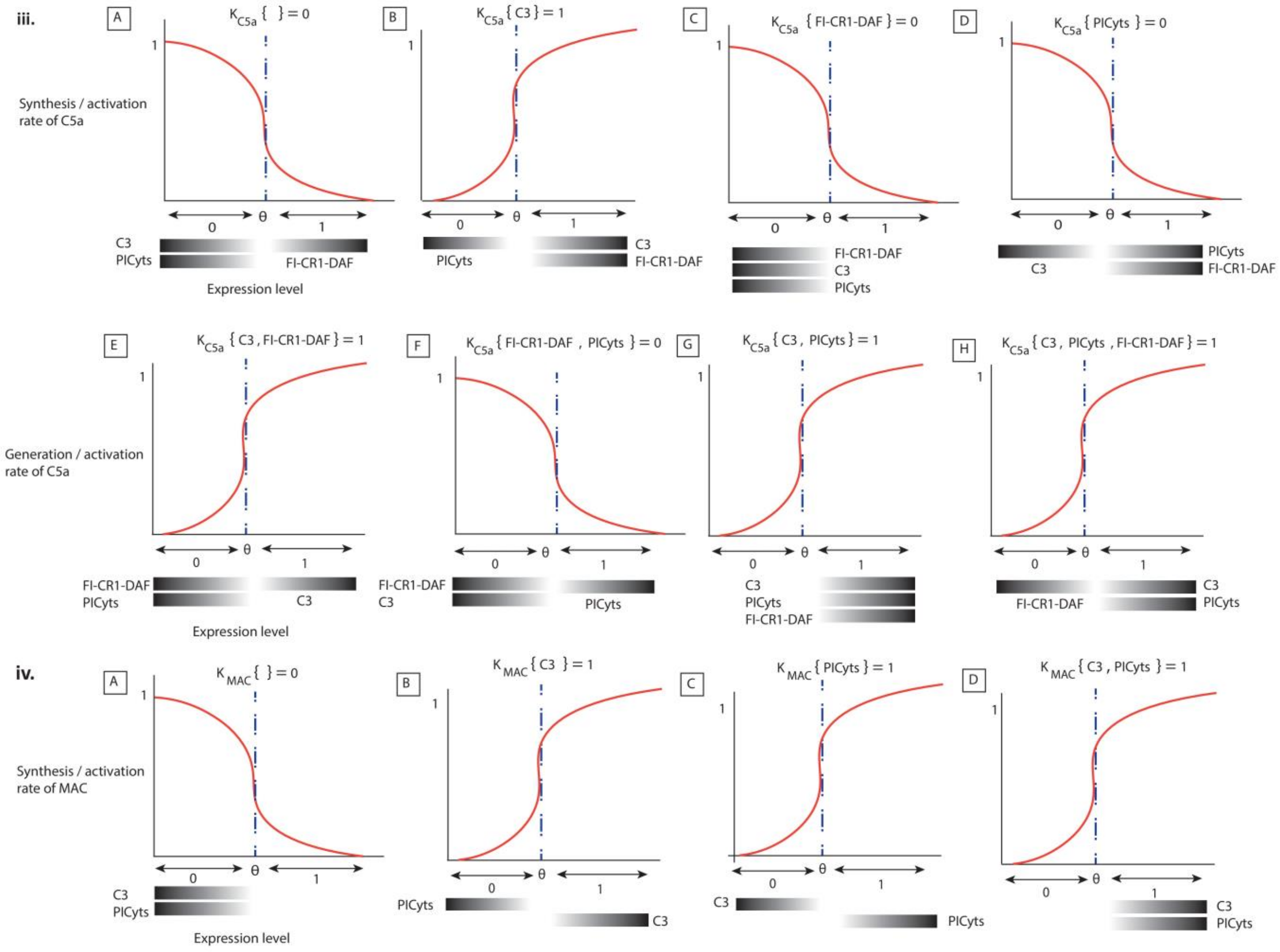

Figure. Dummy tendency graphs. For normal condition, the logical parameters given in Table 2 have been shown through dummy tendency graphs. Along x-axes resource entities and y-axes generation/synthesis level of targeted entities are taken. Logically absence/presence of negative regulator/s as resource indicating it is actually active/inactive for downregulation of the targeted entities [1,2].

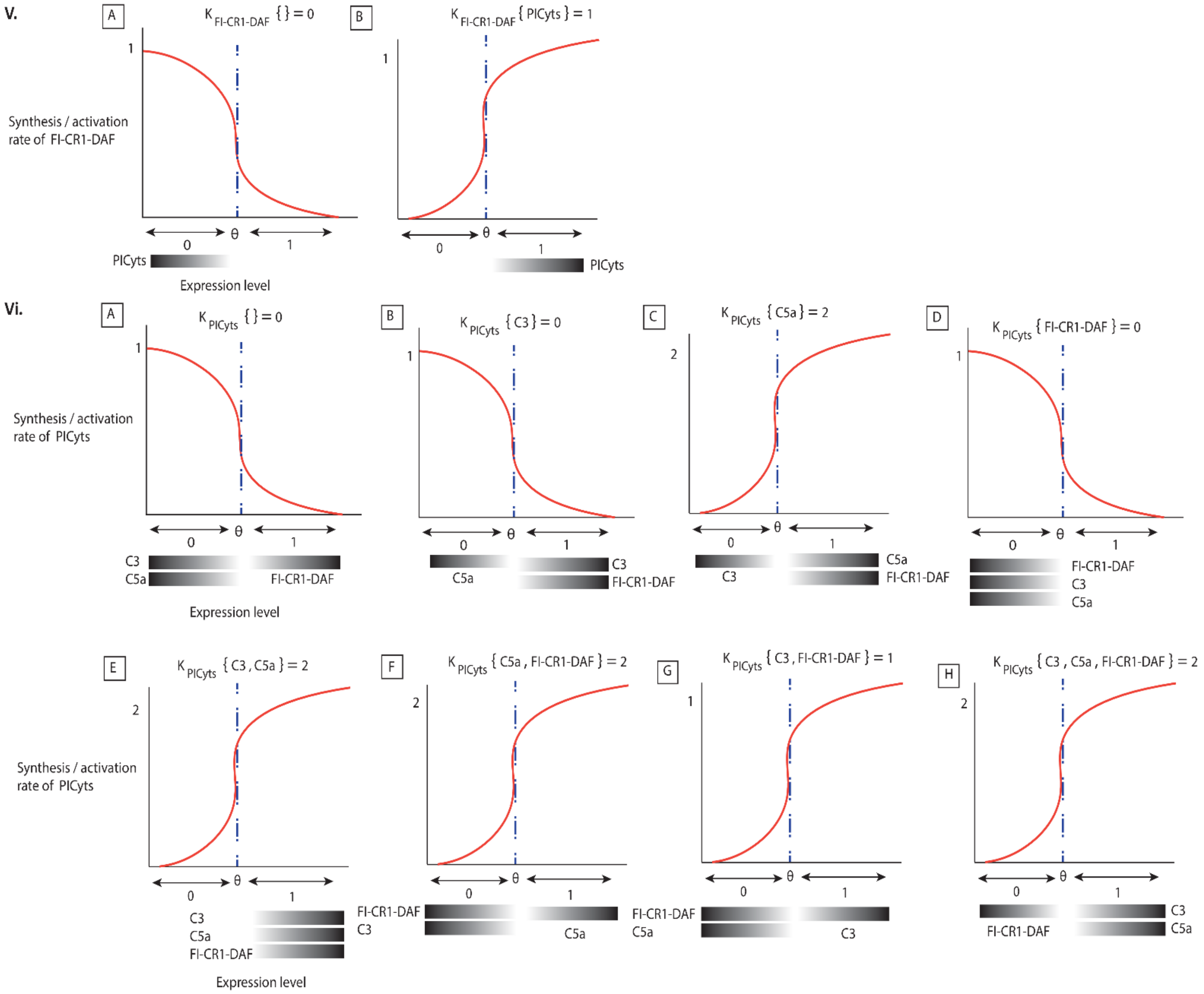

Figure. Dummy tendency graphs. For normal condition, the logical parameters given in Table 2 have been shown through dummy tendency graphs. Along x-axes resource entities and y-axes generation/synthesis level of targeted entities are taken. Logically absence/presence of negative regulator/s as resource indicating it is actually active/inactive for downregulation of the targeted entities [1,2].

References

[1] Paracha RZ, Ahmad J, Ali A, Hussain R, Niazi U, Tareen SHK, et al. Formal modelling of toll like receptor 4 and jak/stat signalling pathways: insight into the roles of socs-1, interferon-β and proinflammatory cytokines in sepsis. PloS one. 2014;9(9):e108466.

[2] Saeed MT, Ahmad J, Baumbach J, Pauling J, Shafi A, Paracha RZ, et al. Parameter estimation of qualitative biological regulatory networks on high performance computing hardware. BMC systems biology. 2018;12(1):1–15.
