# Supplementary material for: Modelling and analysis of the complement system signalling pathways: roles of C3, C5a and pro-inflammatory cytokines in SARS-CoV-2 infection
Source: PeerJ. 2023 Sep 20;11:e15794. doi: 10.7717/peerj.15794 (PMC10517668; doi:10.7717/peerj.15794)
Supplement: Supplemental Information 9 [file peerj-11-15794-s009.pdf]

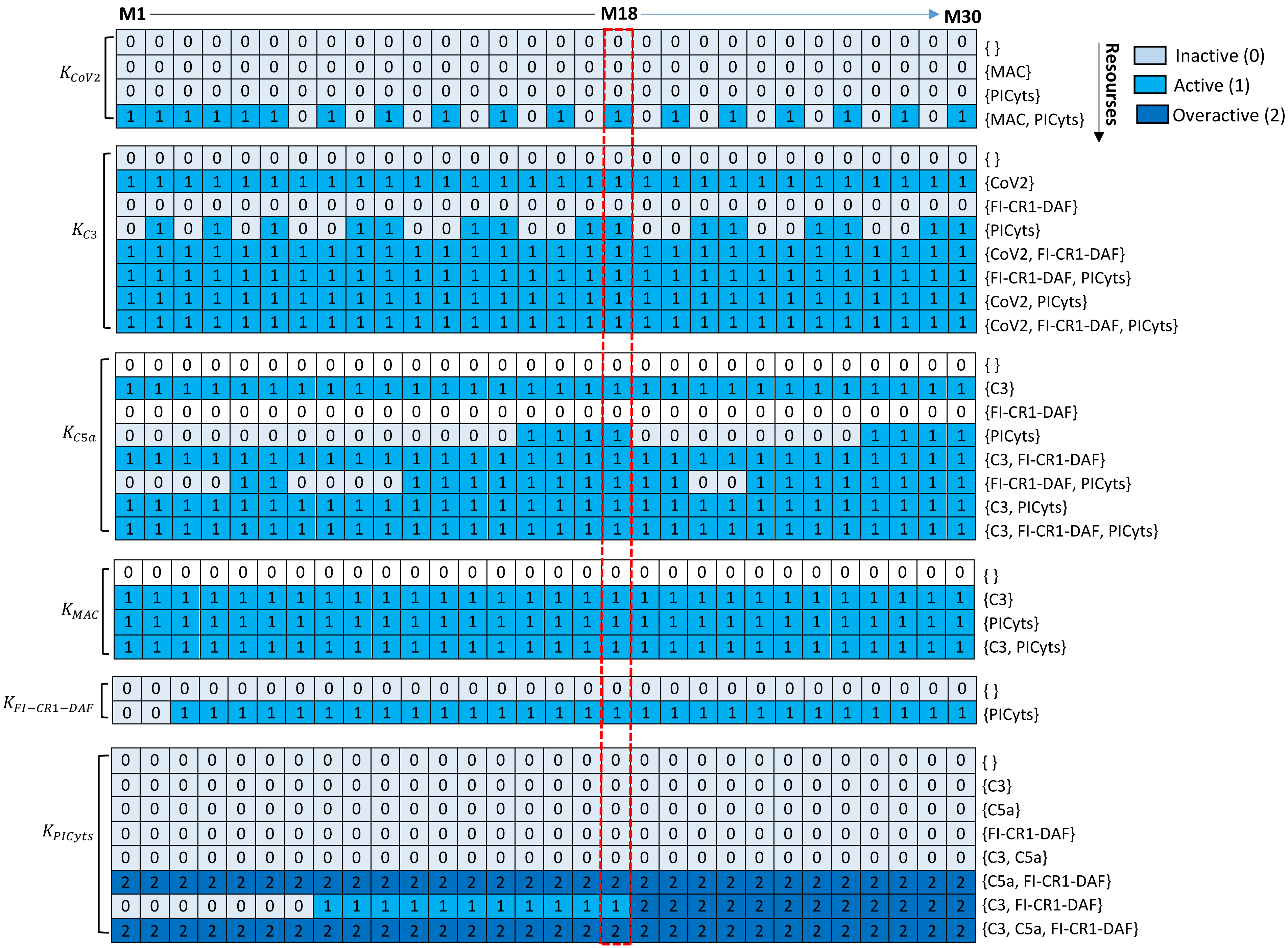

Figure. For case-2 (Intervention in C5a mediated upregulation of PICyts) heatmap representation of logical parameters sets.
