# Supplementary material for: Modelling and analysis of the complement system signalling pathways: roles of C3, C5a and pro-inflammatory cytokines in SARS-CoV-2 infection
Source: PeerJ. 2023 Sep 20;11:e15794. doi: 10.7717/peerj.15794 (PMC10517668; doi:10.7717/peerj.15794)
Supplement: Supplemental Information 5 [file peerj-11-15794-s005.pdf]

Parameter	Resources	M1	M2	M3	M4	M5	M6	M7	M8	M9	M10	M11	M12	M13	M14	M15	M16	M17	M18	M19	M20	M21	M22	M23	M24	M25	M26	M27	M28	M29
K_{CoV2}	{ }	0	0	0	0	0	0	0	0	0	0	0	0	0	0	0	0	0	0	0	0	0	0	0	0	0	0	0	0	0
	{MAC}	0	0	0	0	0	0	0	0	0	0	0	0	0	0	0	0	0	0	0	0	0	0	0	0	0	0	0	0	0
	{PICyts}	0	0	0	0	0	0	0	0	0	0	0	0	0	0	0	0	0	0	0	0	0	0	0	0	0	0	0	0	0
	{MAC, PICyts}	0	1	0	1	0	1	0	1	1	0	1	0	1	0	1	1	0	1	1	1	0	1	0	1	1	0	1	1	1
K_{C3}	{ }	0	0	0	0	0	0	0	0	0	0	0	0	0	0	0	0	0	0	0	0	0	0	0	0	0	0	0	0	0
	{CoV2}	1	1	1	1	1	1	1	1	1	1	1	1	1	1	1	1	1	1	1	1	1	1	1	1	1	1	1	1	1
	{FI-CR1-DAF}	0	0	0	0	0	0	0	0	0	0	0	0	0	0	0	0	0	0	0	0	0	0	0	0	0	0	0	0	0
	{PICyts}	0	0	1	1	0	0	1	1	0	1	1	0	0	1	1	0	1	1	0	1	0	0	1	1	0	1	1	0	1
	{CoV2, FI-CR1-DAF}	1	1	1	1	1	1	1	1	1	1	1	1	1	1	1	1	1	1	1	1	1	1	1	1	1	1	1	1	1
	{FI-CR1-DAF, PICyts}	1	1	1	1	1	1	1	1	1	1	1	1	1	1	1	1	1	1	1	1	1	1	1	1	1	1	1	1	1
	{CoV2, PICyts}	1	1	1	1	1	1	1	1	1	1	1	1	1	1	1	1	1	1	1	1	1	1	1	1	1	1	1	1	1
	{CoV2, FI-CR1-DAF, PICyts}	1	1	1	1	1	1	1	1	1	1	1	1	1	1	1	1	1	1	1	1	1	1	1	1	1	1	1	1	1
K_{C5a}	{ }	0	0	0	0	0	0	0	0	0	0	0	0	0	0	0	0	0	0	0	0	0	0	0	0	0	0	0	0	0
	{C3}	1	1	1	1	1	1	1	1	1	1	1	1	1	1	1	1	1	1	1	1	1	1	1	1	1	1	1	1	1
	{FI-CR1-DAF}	0	0	0	0	0	0	0	0	0	0	0	0	0	0	0	0	0	0	0	0	0	0	0	0	0	0	0	0	0
	{PICyts}	0	0	0	0	0	0	0	0	0	0	0	0	0	0	0	0	0	0	1	1	0	0	0	0	0	0	0	1	1
	{C3, FI-CR1-DAF}	1	1	1	1	1	1	1	1	1	1	1	1	1	1	1	1	1	1	1	1	1	1	1	1	1	1	1	1	1
	{FI-CR1-DAF, PICyts}	0	0	0	0	0	0	0	0	1	1	1	0	0	0	0	1	1	1	1	1	0	0	0	0	1	1	1	1	1
	{C3, PICyts}	1	1	1	1	1	1	1	1	1	1	1	1	1	1	1	1	1	1	1	1	1	1	1	1	1	1	1	1	1
	{C3, FI-CR1-DAF, PICyts}	1	1	1	1	1	1	1	1	1	1	1	1	1	1	1	1	1	1	1	1	1	1	1	1	1	1	1	1	1
K_{MAC}	{ }	0	0	0	0	0	0	0	0	0	0	0	0	0	0	0	0	0	0	0	0	0	0	0	0	0	0	0	0	0
	{C3}	1	1	1	1	1	1	1	1	1	1	1	1	1	1	1	1	1	1	1	1	1	1	1	1	1	1	1	1	1
	{PICyts}	1	1	1	1	1	1	1	1	1	1	1	1	1	1	1	1	1	1	1	1	1	1	1	1	1	1	1	1	1
	{C3, PICyts}	1	1	1	1	1	1	1	1	1	1	1	1	1	1	1	1	1	1	1	1	1	1	1	1	1	1	1	1	1
$K_{FI-CR1-DAF}$	{ }	0	0	0	0	0	0	0	0	0	0	0	0	0	0	0	0	0	0	0	0	0	0	0	0	0	0	0	0	0
	{PICyts}	0	0	0	0	1	1	1	1	1	1	1	1	1	1	1	1	1	1	1	1	1	1	1	1	1	1	1	1	1
K_{PICyts}	{ }	0	0	0	0	0	0	0	0	0	0	0	0	0	0	0	0	0	0	0	0	0	0	0	0	0	0	0	0	0
	{C3}	0	0	0	0	0	0	0	0	0	0	0	0	0	0	0	0	0	0	0	0	0	0	0	0	0	0	0	0	0
	{C5a}	2	2	2	2	2	2	2	2	2	2	2	2	2	2	2	2	2	2	2	2	2	2	2	2	2	2	2	2	2
	{FI-CR1-DAF}	0	0	0	0	0	0	0	0	0	0	0	0	0	0	0	0	0	0	0	0	0	0	0	0	0	0	0	0	0
	{C3, C5a}	2	2	2	2	2	2	2	2	2	2	2	2	2	2	2	2	2	2	2	2	2	2	2	2	2	2	2	2	2
	{C5a, FI-CR1-DAF}	2	2	2	2	2	2	2	2	2	2	2	2	2	2	2	2	2	2	2	2	2	2	2	2	2	2	2	2	2
	{C3, FI-CR1-DAF}	0	0	0	0	0	0	0	0	0	0	0	1	1	1	1	1	1	1	1	1	2	2	2	2	2	2	2	2	2
	{C3, C5a, FI-CR1-DAF}	2	2	2	2	2	2	2	2	2	2	2	2	2	2	2	2	2	2	2	2	2	2	2	2	2	2	2	2	2

Resourses

Inactive (0)
Active (1)
Overactive (2)

Figure: For pathogenic condition heatmap representation of logical parameters sets.
