# Supplementary material for: Modelling and analysis of the complement system signalling pathways: roles of C3, C5a and pro-inflammatory cytokines in SARS-CoV-2 infection
Source: PeerJ. 2023 Sep 20;11:e15794. doi: 10.7717/peerj.15794 (PMC10517668; doi:10.7717/peerj.15794)
Supplement: Supplemental Information 12 [file peerj-11-15794-s012.pdf]

Modelling and analysis of complement system signalling pathways: Roles of C3, C5a and pro-inflammatory cytokines in SARS-CoV-2 infection

Didar Murad[1], Rehan Zafar Paracha[1*], Muhammad Tariq Saeed[1], Jamil Ahmad[2], Ammar Mushtaq[1], and Maleeha Humayun[1]

[1]**School of Interdisciplinary Engineering and Sciences/Department of Sciences/National University of Sciences and Technology, H-12, Islamabad, Pakistan**
[2]**Department of Computer Science and Information Technology/University of Malakand, Chakdara, Khyber Pakhtunkhwa, Pakistan**

Corresponding author:
Rehan Zafar Paracha[1*]

Email address: rehan@sines.nust.edu.pk

The Computational Tree Logic (CTL) formalism fundamentals (Paracha, 2015; Chabrier and Fages, 2003)

The CTL formula is defined inductively with:

- A finite state of propositional variables: $\{v_i\}$.
- Logical connectives: $\top$ (boolean true), $\bot$ (boolean false), $\neg$ (negation), $\wedge$ (logical AND), $\vee$ (logical OR), $\Rightarrow$ (implication).
- Temporal connectives: AX, EX, AG, EG, AU, EU, AF, EF.
- Rules of formation: v_i, $\top$, $\bot$ are formulae. If ϕ_1 and ϕ_2 are proportional logic formulae then $(\neg\phi_1), (\phi_1 \wedge \phi_2), (\phi_1 \vee \phi_2), (\phi_1 \Rightarrow \phi_2), AX\phi_1,\ EX\phi_1,\ A[\phi_1 U \phi_2],\ E[\phi_1 U \phi_2]$,
  $AG\phi_1, EG\phi_1, AF\phi_1,\ EF\phi_1$ are formulae.
  The logical connectives are the classical ones: false, true, not, and, or implication. All the temporal connectives are pairs of symbols. The first element of the pair is A or E. The second one is temporal operator as X, F, G or U.
  Meaning of the Connectives:
  – A : along All paths
  – E : along at least one path (there Exist)
  – X : neXt state
  – F (also written as $'\diamond'$): some Future state
  – G (also written as $'\Box'$) : all future states (Globally)
  – U : Until
  All the CTL connectives are equivalent to a combination of a set of six of them, for example $\{\bot, \neg, \wedge$, AF, EU, EX$\}$. So, all the formulas can be written with this set of connectives using the following equivalences.
  Equivalences between CTL Formulae
  1. $\neg AF\phi \equiv EG\neg\phi$
  2. $\neg EF\phi \equiv AG\neg\phi$

3. $\neg AX\phi \equiv EX\neg\phi$
4. $AF\phi \equiv A[\top \cup \phi]$
5. $EF\phi \equiv E[\top \cup \phi]$
6. $A[p \cup q] \equiv \neg(E[\neg qU(\neg p \wedge \neg q)] \vee EG\neg q)$

Establishment of CTL:

- State formulae syntax as ϕ := true v_i, $\phi_1 \wedge \Phi_2$, $\neg\phi_1$, Eα, Aα, where: ϕ_1, ϕ_2 are state formulae, α : is path formula.
- Path formulae syntax as α:= ϕ, Xα_1, α_1Uα_2, Fα_1, Gα_1, where: ϕ is state formula, α_1 α_2: are path formulae.
- We can write path formulae as state formula for development of CTL as $X\phi_1$, ϕ_1Uϕ_2, $F\phi_1$, $G\phi_1$.
- So, with the help of above governing rules we can write CTLs, some examples of legal and illegal CTLs are given as
  – Legal CTL formulae: EFv_1, EFAGv_1, AXv_2, AFv_1 $\wedge$ AGv_2, A(v_1 U (FGv_2)).
  – Illegal CTL formulae: AFGv_1, Av_1, EGFv_i, A(Fv_1 $\wedge$ Gv_2), A(v_1 U (Gv_2)).

REFERENCES

Chabrier, N. and Fages, F. (2003). Symbolic model checking of biochemical networks. In *International Conference on Computational Methods in Systems Biology*, pages 149–162. Springer.

Paracha, R. Z. (2015). Modelling of tlr4 and jak/stat signalling pathways and protein-protein interaction studies to examine the pathophysiology of sepsis. phd thesis. *National University of Sciences and Technology (NUST) Islamabad, Pakistan*.
