# Supplementary material for: Modelling and analysis of the complement system signalling pathways: roles of C3, C5a and pro-inflammatory cytokines in SARS-CoV-2 infection
Source: PeerJ. 2023 Sep 20;11:e15794. doi: 10.7717/peerj.15794 (PMC10517668; doi:10.7717/peerj.15794)
Supplement: Supplemental Information 14 [file peerj-11-15794-s014.pdf]

Modelling and analysis of complement system signalling pathways: Roles of C3, C5a and pro-inflammatory cytokines in SARS-CoV-2 infection

Didar Murad[1], Rehan Zafar Paracha[1*], Muhammad Tariq Saeed[1], Jamil Ahmad[2], Ammar Mushtaq[1], and Maleeha Humayun[1]

[1]**School of Interdisciplinary Engineering and Sciences/Department of Sciences/National University of Sciences and Technology, H-12, Islamabad, Pakistan**
[2]**Department of Computer Science and Information Technology/University of Malakand, Chakdara, Khyber Pakhtunkhwa, Pakistan**

Corresponding author:
Rehan Zafar Paracha[1*]

Email address: rehan@sines.nust.edu.pk

Description of logical parameters with relevant evidences

For the entities in the Biological Regulatory Network (BRN) below is the description of logical parameters with experimental evidences. Based on well established observations in literature, some logical parameters are provided with fixed single values. Targeted entity is considered to be inhibited/inactivated, due to absence of activator/s or presence of inhibitor/s (Paracha et al., 2014; Saeed et al., 2018).

For normal condition, logical parameters sets (models) are computed, the algorithm of NuSMV is provided in File S2. The parameters sets are shown via heatmap in Figure S1. Here we used model-14.

Target: CoV2
Resources include: Inhibitors MAC and PICyts

1. $K_{CoV2}\{\} = 0$
   - **Description:** MAC and PICyts active for inactivation of CoV2.
   - **Evidence:** Experimental studies are conducted that are associated with suppression of COVID-19. PICyts caused pro-inflammatory response for lowering SARS-CoV-2 titre (Carvelli et al., 2020; Kim et al., 2020; Ram Kumar Pandian et al., 2020). MAC (C5b-9) via lysis can inhibits SARS-CoV-2 (Chouaki Benmansour et al., 2021). MAC also stimulates inflammatory response against the pathogen (Garred et al., 2021; Bakshi et al., 2020; Hovland et al., 2015).
   - **SMBioNet input values:** Minimum and maximum expression level provided as 0 and 1.
   - **SMBioNet output value and inference:** The output value ″0″ infer that due to active inhibitory entities MAC and PICyts CoV2 would be inactive.
2. $K_{CoV2}\{MAC\} = 0$
   - **Description:** MAC is inactive and PICyts is active for CoV2.
   - **Evidence:** For some evidences with respect to active PICyts for the inhibation of CoV2 (see point-1). Additionally, active PICyts activate Inflammatory cells (ICs) which phagocytosis the virus directly in begin phase of innate response, clear the pathogen by promoting inflammation

(Noris et al., 2020). PICyts activate ICs which further activate IFN$\alpha \backslash \beta$ to inhibit the pathogen (Shemesh et al., 2021; Zhang et al., 2021; Yang et al., 2021).

- **SMBioNet input values:** Minimum and maximum expression level provided as 0 and 1.
- **SMBioNet output value and inference:** The output value $''0''$ implies that CoV2 is inhibited due to the presence of PICyts.

3. $K_{CoV2}\{PICyts\} = 0$

- **Description:** PICyts is inactive and MAC is active for CoV2.
- **Evidence:** Some evidences with respect to active MAC for inhibition of CoV2 are maintained in point-1. Moreover, active MAC suppresses CoV2 as It forms cytotoxic pores on the surface of pathogens. MAC punches a hole through the plasma membrane of the target cell, killing the pathogen and causes lysis of the pathogen. (Polycarpou et al., 2020; Shibabaw et al., 2020). It plays a role in host defense processes through its ability to kill the virus and to promote inflammation by stimulating inflammatory cells (Xie et al., 2020).
- **SMBioNet input values:** Minimum and maximum expression level provided as 0 and 1.
- **SMBioNet output value and inference:** The output value $''0''$ implies that CoV2 is inactivated due to active MAC.

4. $K_{CoV2}\{MAC, PICyts\} = 0$

- **Description:** MAC and PICyts are inactive for CoV2.
- **Evidence:** PICyts is inactive as FI-CR1-DAF mediated activated C3a-C3aR and/or C5a-C5aR axis-es via ICs inactivate PICyts. Inflammatory cells produce IFN$\alpha \backslash \beta$, which inactivated PICyts (Chalise et al., 2013). Moreover, MAC is inactive as IFN$\alpha \backslash \beta$ inhibit MAC (Figure 1). The trajectory of inhibation followed as IFN$\alpha \backslash \beta$ mediated PICyts and/or IFNγ, ICs and lymphocytes which synthesise and activate terminal complement protein C5 (Lubbers et al., 2017) suppress MAC.
- **SMBioNet input values:** Minimum and maximum expression level provided as 0 and 1.
- **SMBioNet output value and inference:** The output value $''0''$ deduced that CoV2 would be inactivated in the absence of downregulators MAC and PICyts due to absence of upregulators.

Target: C3
Resources include: Activators CoV2 and PICyts, Inhibitor FI-CR1-DAF

5. $K_{C3}\{\} = 0$

- **Description:** CoV2 and PICyts are inactive while FI-CR1-DAF is active for C3.
- **Evidence:** Due to unavailability of CoV2 and PICyts, C3 unable to upregulate. Due to presence of FI-CR1-DAF the C3 can be suppressed. CR1 and DAF inhibit the formation of C3-convertase (Zewde et al., 2016; Thurman and Renner, 2011; Ahmad et al., 2003). Moreover, DAF decay the C3-convertase (Bansal et al., 2022). FI can bind with C3b and inhibit C3-convertase assembly (Bansal et al., 2022; Shinjyo et al., 2021). FI-CR1-DAF mediated C3-convertase inactivates C3 (Figure 1).
- **SMBioNet input values:** Minimum and maximum expression level provided as 0 and 1.
- **SMBioNet output value and inference:** The value $''0''$ as output infer that in the absence of CoV2 and PICyts as activators and presence of FI-CR1-DAF as inhibator C3 would be downregulated.

6. $K_{C3}\{CoV2\} = 1$

- **Description:** CoV2 and FI-CR1-DAF are active, PICyts is inactive for C3.

- **Evidence:** The SARS-CoV-2 bind and activate different initial complements of the complement cascade, ultimately triggers C3 (Shinjyo et al., 2021; Jodele and Köhl, 2021; Ng and Powell, 2021; Detsika and Lianos, 2021; Dijkstra et al., 2019). Moreover, it is experimentally observed that C3 is highly expressed due to SARS-CoV-2 (Henry et al., 2021; Yan et al., 2021).
- **SMBioNet input values:** 1 as fixed value.
- **SMBioNet output value and inference:** The value $''1''$ as output implies that due to loss of active negative regulatory complex FI-CR1-DAF and presence of CoV2 the C3 would be activated.

7. $K_{C3}\{FI-CR1-DAF\} = 0$

- **Description:** FI-CR1-DAF, CoV2 and PICyts are inactive.
- **Evidence:** Due to absence of CoV2 and PICyts, C3 remain inactivated. It is observed that the concentration levels of FI-CR1-DAF are low during SARS-CoV-2 infection. We can assume FI-CR1-DAF is inactive for C3 (Kisserli et al., 2021; Alosaimi et al., 2021).
- **SMBioNet input values:** Minimum and maximum expression level provided as 0 and 1.
- **SMBioNet output value and inference:** The value $''0''$ as output implies that due to unavailability of activating factors CoV2 and PICyts C3 would be inactivated.

8. $K_{C3}\{PICyts\} = 1$

- **Description:** PICyts, FI-CR1-DAF are active and CoV2 is inactive for C3.
- **Evidence:** Active PICyts can increase C3 expression and active FI-CR1-DAF inhibits C3 (Dos Santos et al., 2017). The C3 production is positive correlate with IL-6. IL-6 concentration level reported high in PICyts storm, at the same time C3 level reported high (Aljwaid et al., 2021). Moreover, the trajectory mediated PICyts and ICS denoting activation of C3 as shown in CS signalling pathways (Figure 1).
- **SMBioNet input values:** Minimum and maximum expression level provided as 0 and 1.
- **SMBioNet output value and inference:** The output value $''1''$ implies that activating factor PICyts and loss of active inhibator FI-CR1-DAF would activate C3.

9. $K_{C3}\{CoV2,\ FI-CR1-DAF\} = 1$

- **Description:** CoV2 is active, FI-CR1-DAF and PICyts are inactive for C3.
- **Evidence:** The SARS-CoV-2 load can increase the concentration level of C3. FI-CR1-DAF is inactive for C3 (Kisserli et al., 2021; Alosaimi et al., 2021).
- **SMBioNet input values:** Minimum and maximum expression level provided as 0 and 1.
- **SMBioNet output value and inference:** The output value $''1''$ implies that the availability of CoV2 would activate C3.

10. $K_{C3}\{FI-CR1-DAF,\ PICyts\} = 1$

- **Description:** PICyts is active, FI-CR1-DAF and CoV2 are inactive for C3.
- **Evidence:** Followed point-8.
- **SMBioNet input values:** Minimum and maximum expression level provided as 0 and 1.
- **SMBioNet output value and inference:** The output value $''1''$ infer that the positive regulator PICyts would activate C3.

11. $K_{C3}\{CoV2,\ PICyts\} = 1$

- **Description:** CoV2, PICyts and FI-CR1-DAF are active for C3.

- **Evidence:** Some references are followed from point-8 with respect to activation of C3 due to PICyts presence. Additionally, higher titres of SARS-CoV-2 and PICyts recruit more innate immune cells (eg. macrophages and neutrohils) and acquired immune cells (T-cells). The positive loop between the Cells and PICyts (Risitano et al., 2020) implies both entities are directly correlated. PICyts mediated ICS upregulate the C3. The CoV2 induced complement system pathways lead to production of C3 (Figure 1).
- **SMBioNet input values:** Minimum and maximum expression level provided as 0 and 1.
- **SMBioNet output value and inference:** The output value ″1″ infer that the C3 would be generated due to existence of activating factors CoV2 and PICyts and loss of active inhibator FI-CR1-DAF.

12. $K_{C3}\{CoV2,\ PICyts,\ FI-CR1-DAF\} = 1$
    - **Description:** CoV2, PICyts are active and FI-CR1-DAF is inactive for C3.
    - **Evidence:** Some relevant references are to be followed from point-8 with respect to activation of C3 due to PICyts presence. Additionally, higher titres of SARS-CoV-2 and overexpressed PICyts have been recruited more innate immune cells (eg. macrophages and neutrohils) and acquired immune cells (T-cells). Positive loop between ICs and PICyts (Fan et al., 2021; Risitano et al., 2020) implies both entities are directly correlated. PICyts mediated ICS upregulate C3.
    - **SMBioNet input values:** 1 as fixed value.
    - **SMBioNet output value and inference:** The output value ″1″ implies that the C3 would be activated due to the presence of activators CoV2 and PICyts.

Target: C5a
Resources include: Activators C3 and PICyts, Inhibitor FI-CR1-DAF

13. $K_{C5a}\{\} = 0$
    - **Description:** C3 and PICyts are inactive and FI-CR1-DAF is active for C5a.
    - **Evidence:** C5-convertase cleaved C5 into C5a and C5b. FI-CR1-DAF mediated C5-convertase inhibit C5a (Figure 1). Moreover, during SARS-CoV-2 recurrent infection it may be possible FI-CR1-DAF expressed high, and assumed FI-CR1-DAF is active with the low response level of PICyts (see Figure 9E).
    - **SMBioNet input values:** Minimum and maximum expression level provided as 0 and 1.
    - **SMBioNet output value and inference:** The output value ″0″ infer that when FI-CR1-DAF be in activate state then C5a would be degenerated.

14. $K_{C5a}\{C3\} = 1$
    - **Description:** C3, FI-CR1-DAF are active and PICyts is inactive for C5a.
    - **Evidence:** C3 is activated by CoV2 via C3-convertase, which is activated though C1 of classical pathway, MBL of lectin pathway and C3 of alternative pathways (Noris et al., 2020). Experimental studies implies concentration level of C5a is reported high in mild, moderate and severe patients (Henry et al., 2021; Yan et al., 2021; Showers et al., 2021). Expression levels of FI-CR1-DAF are low during SARS-CoV-2 infection and we assume FI-CR1-DAF is inactive for C5a (Kisserli et al., 2021; Alosaimi et al., 2021).
    - **SMBioNet input values:** 1 as fixed value.
    - **SMBioNet output value and inference:** The output value ″1″ implies that when C3 is in active state then C5a would be activated even in the presence of FI-CR1-DAF.

15. $K_{C5a}\{FI-CR1-DAF\} = 0$
    - **Description:** FI-CR1-DAF, C3 and PICyts are inactive for C5a.

- **Evidence:** It is necessary the presence of activating factors C3 and PIcyts for upregulation of C5a.
- **SMBioNet input values:** Provided 0 as fixed value.
- **SMBioNet output value and inference:** The output value $''0''$ implies that due to unavailability of activators, the C5a would remain suppressed.

16. $K_{C5a}\{PICyts\} = 1$

- **Description:** PICyts, FI-CR1-DAF are active and C3 is inactive for C5a.
- **Evidence:** Positive loop exist between cytokine storm and overactivated inflammatory cells macrophages and neutrophils (Figure 1). PICyts mediated inflammatory cells can induce C5a Atri et al. (2018).
- **SMBioNet input values:** Minimum and maximum expression level provided as 0,1
- **SMBioNet output value and inference:** The output value $''1''$ infer that C5a would be induced due to the presence of activator PICyts. Even the active FI-CR1-DAF not inhibit C5a, it may be due to loss of FI-CR1-DAF.

17. $K_{C5a}\{C3,\ FI-CR1-DAF\} = 1$

- **Description:** C3 is active, FI-CR1-DAF and PICyts are inactive for C5a.
- **Evidence:** Followed point-14.
- **SMBioNet input values:** Minimum and maximum expression level provided as 0 and 1.
- **SMBioNet output value and inference:** The output value $''1''$ infer that when C3 is in active state then C5a would be activated.

18. $K_{C5a}\{FI-CR1-DAF,\ PICyts\} = 1$

- **Description:** FI-CR1-DAF, C3 are inactive,and PICyts active for C5a.
- **Evidence:** Followed point-16.
- **SMBioNet input values:** Minimum and maximum expression level provided as 0 and 1.
- **SMBioNet output value and inference:** The output value $''1''$ implies that C5a would be induced due to the presence of activator PICyts.

19. $K_{C5a}\{C3,\ PICyts\} = 1$

- **Description:** C3 and PICyts and FI-CR1-DAF are active for C5a.
- **Evidence:** Followed point-14 and point-16.
- **SMBioNet input values:** Minimum and maximum expression level provided as 0 and 1.
- **SMBioNet output value and inference:** The output value $''1''$ deduced that C5a would be induced due to the presence of activators C3 and PICyts even the active FI-CR1-DAF not downregulate C5a.

20. $K_{C5a}\{C3,\ PICyts,\ FI-CR1-DAF\} = 1$

- **Description:** C3, PICyts are active and FI-CR1-DAF is inactive for C5a.
- **Evidence:** The active C3 leads to activation of C5a. C3 is activated by CoV2 via C3-convertase, which is activated though C1 of classical pathway and MBL of lectin pathway (Noris et al., 2020). During SARS-CoV-2 infection, massive production of PICyts result in overstimulation of inflammatory cells, which leads to induction of C5a (Figure 1).
- **SMBioNet input values:** Provided 1 as fixed value.
- **SMBioNet output value and inference:** The output value $''1''$ infer that C5a would be induced due to the presence of activators C3 and PICyts.

Target: MAC
Resources include: Activators C3 and PICyts

21. $K_{MAC}\{\} = 0$

- **Description:** C3 and PICyts are inactive for MAC.
- **Evidence:** MAC is unable to induce due to absence of activating entities.
- **SMBioNet input values:** Minimum and maximum expression level provided as 0 and 1.
- **SMBioNet output value and inference:** The output value ″0″ implied that due to unavailability of activators MAC remain inactive.

22. $K_{MAC}\{C3\} = 1$

- **Description:** C3 is active and PICyts is inactive for MAC.
- **Evidence:** The presence of C3 is an initial factor for formation of terminal complement complex (TCC) MAC. Due to active C3 followed by triggering and cleaving mechanism of complement entities lead to the formation of MAC. The complete scenario of MAC induction shown in Figure 1.
- **SMBioNet input values:** 1 (fixed value).
- **SMBioNet output value and inference:** The output value ″1″ deduced that MAC would be generated due to the presence of upregulator C3.

23. $K_{MAC}\{PICyts\} = 1$

- **Description:** PICyts is active and C3 is inactive for MAC.
- **Evidence:** Positive loop Risitano et al. (2020) exist between cytokine storm and overactivated inflammatory cells macrophages and neutrophils (Figure 1). PICyts stimulate ICs which activate C5 ultimately formation of active MAC.
- **SMBioNet input values:** Minimum and maximum expression level provided as 0 and 1.
- **SMBioNet output value and inference:** The output value ″1″ infer that MAC would be induced due to the presence of activator PICyts.

24. $K_{MAC}\{C3, PICyts\} = 1$

- **Description:** C3 and PICyts are active for MAC.
- **Evidence:** Followed point-22 and point-23.
- **SMBioNet input values:** Provided 1 as fixed value.
- **SMBioNet output value and inference:** The output value ″1″ implies that MAC would be produced due to the presence of activators C3 and PICyts.

Target: FI-CR1-DAF
Resources include: Activator PICyts

25. $K_{FI-CR1-DAF}\{\} = 0$

- **Description:** PICyts is inactive for FI-CR1-DAF.
- **Evidence:** FI-CR1-DAF remain inactive due to the absence of activator PICyts.
- **SMBioNet input values:** Minimum and maximum expression level provided as 0 and 1
- **SMBioNet output value and inference:** The output value ″0″ infer that FI-CR1-DAF would be inactivated due to unavailability of activating factor PICyts.

26. $K_{FI-CR1-DAF}\{PICyts\} = 1$

- **Description:** PICyts is active for FI-CR1-DAF.

- **Evidence:** CR1 and DAF bind with C3b (Forneris et al., 2016), which are produced in a limited level due to normal stimulation of PICyts during SARS-CoV-2 infection.
- **SMBioNet input values:** Minimum and maximum expression level provided as 0 and 1.
- **SMBioNet output value and inference:** The output value ″1″ implies that due the presence of PICyts, FI-CR1-DAF would be activated.

Target: PICyts
Resources include: Activators C3 and C5a, inactivator FI-CR1-DAF

27. $K_{PICyts}\{\} = 0$

- **Description:** C3, C5a are inactive and FI-CR1-DAF is active for PICyts.
- **Evidence:** FI-CR1-DAF directly inhibits C3-convertase and C5-convertase (Thurman and Renner, 2011; Poppelaars et al., 2018; Chen et al., 2022) then via C3a-C3aR and C5a-C5aR axises able to suppress the generation of inflammatory cells (macrophages and neutrophils) and PICyts. In SARS-CoV-2 induced complement system pathways (Figure 1) positive loop (Hembram, 2021; Risitano et al., 2020; Fan et al., 2021) between cytokine storm and overactivated inflammatory cells implies there is direct correlation exist between inflammatroy cells and PICyts.
- **SMBioNet input values:** Minimum and maximum expression level provided as 0 and 2.
- **SMBioNet output value and inference:** The output value ″0″ infer that due to availability of negative regulator FI-CR1-DAF, PICyts production would be suppressed.

28. $K_{PICyts}\{C3\} = 0$

- **Description:** C3, FI-CR1-DAF are active and C5a is inactive for PICyts.
- **Evidence:** FI-CR1-DAF directly inhibits C3-convertase and C5-convertase Thurman and Renner (2011); Poppelaars et al. (2018); Chen et al. (2022) then via C3a-C3aR and C5a-C5aR axises able to suppress the generation of inflammatory cells and PICyts. In SARS-CoV-2 induced complement system pathways (Figure 1) positive loop (Hembram, 2021; Risitano et al., 2020; Fan et al., 2021) between cytokine storm and overactivated inflammatory cells implies there is direct correlation exist between inflammatroy cells and PICyts.
- **SMBioNet input values:** Minimum and maximum expression level provided as 0 and 2.
- **SMBioNet output value and inference:** The output value ″0″ implies that due to presence of inhibator FI-CR1-DAF, PICyts would be inactivated even the activator C3 actively involved in the induction of PICyts.

29. $K_{PICyts}\{C5a\} = 2$

- **Description:** C5a, FI-CR1-DAF are active and C3 is inactive for PICyts.
- **Evidence:** In COVID-19 due to overexpression of C5a, PICyts found overactivated and caused cytokine storm (Hembram, 2021; Fan et al., 2021).
- **SMBioNet input values:** Provide 2 as fixed value.
- **SMBioNet output value and inference:** The output value as ″2″ deduced that due to the presence of activator C5a and even existence of inhibator FI-CR1-DAF, PICyts would be overactivated.

30. $K_{PICyts}\{FI-CR1-DAF\} = 0$

- **Description:** FI-CR1-DAF, C3, and C5a are inactive for PICyts.
- **Evidence:** Presence of active C3 and C5a are necessary for the induction of PICyts Fan et al. (2021).
- **SMBioNet input values:** Minimum and maximum expression level provided as 0 and 2.
- **SMBioNet output value and inference:** The output value as ″0″ implies that due to absence of any activators C3 and C5a, PICyts would not be activated.

31. $K_{PICyts}\{C3,\ C5a\} = 2$

- **Description:** C3, C5a, and FI-CR1-DAF are active for PICyts.
- **Evidence:** Active C3 and C5a mediated leukocytes and lymphocytes produced massive production of PICyts (Fan et al., 2021).
- **SMBioNet input values:** Provide 2 as fixed value.
- **SMBioNet output value and inference:** The output value $''2''$ infer that due to the availability of activators C3 and C5a, PICyts can reached to higher concentration level even the FI-CR1-DAF is actively evolved for downregulation of PICyts.

32. $K_{PICyts}\{C5a,\ FI-CR1-DAF\} = 2$

- **Description:** C5a is active, FI-CR1-DAF and C3 are inactive for PICyts.
- **Evidence:** Active C5a produce PICyts. During SARS-CoV-2 infection, C5a massively generates PICyts (cytokine storm) (Hembram, 2021; Fan et al., 2021).
- **SMBioNet input values:** Minimum and maximum expression level provided as 0 and 2.
- **SMBioNet output value and inference:** The output value $''2''$ infer that due to the availability of activator C5a, PICyts would be overactivated.

33. $K_{PICyts}\{C3,\ FI-CR1-DAF\} = 1$

- **Description:** C3 is active, FI-CR1-DAF and C5a are inactive for PICyts.
- **Evidence:** PICyts is produced by active C3 as C3 via C3a-C3aR axis mediated inflammatory cells generate PICyts (Fan et al., 2021).
- **SMBioNet input values:** Minimum and maximum expression level provided as 0 and 2.
- **SMBioNet output value and inference:** The output value $''1''$ implies that due to presence of C3, PICyts would be activated.

34. $K_{PICyts}\{C3,\ C5a,\ FI-CR1-DAF\} = 2$

- **Description:** C3 and C5a are active, FI-CR1-DAF is inactive for PICyts.
- **Evidence:** Active C3 and C5a can trigger PICyts. C3 and C5a mediated leukocytes and lymphocytes overactivate PICyts (Fan et al., 2021).
- **SMBioNet input values:** Provided 2 as fixed value.
- **SMBioNet output value and inference:** The output value $''2''$ implies that due to the presence of activators C3 and C5a, PICyts would reached to a higher expression level.

REFERENCES

Ahmad, S. R., Lidington, E. A., Ohta, R., Okada, N., Robson, M. G., Davies, K. A., Leitges, M., Harris, C. L., Haskard, D. O., and Mason, J. C. (2003). Decay-accelerating factor induction by tumour necrosis factor-α, through a phosphatidylinositol-3 kinase and protein kinase c-dependent pathway, protects murine vascular endothelial cells against complement deposition. *Immunology*, 110(2):258–268.

Aljwaid, H. O., Ghali, T. J., AL-HISNAWI, A., and Abass, K. S. (2021). Complement component c3 and c4 in patients covid-19 induced cytokine released storm. *Pakistan Journal of Medical and Health Sciences*, 15(3):1211–1214.

Alosaimi, B., Mubarak, A., Hamed, M. E., Almutairi, A. Z., Alrashed, A. A., AlJuryyan, A., Enani, M., Alenzi, F. Q., and Alturaiki, W. (2021). Complement anaphylatoxins and inflammatory cytokines as prognostic markers for covid-19 severity and in-hospital mortality. *Frontiers in Immunology*, 12:2298.

Atri, C., Guerfali, F. Z., and Laouini, D. (2018). Role of human macrophage polarization in inflammation during infectious diseases. *International journal of molecular sciences*, 19(6):1801.

Bakshi, S., Cunningham, F., Nichols, E.-M., Biedzka-Sarek, M., Neisen, J., Petit-Frere, S., Bessant, C., Bansal, L., Peletier, L. A., Zamuner, S., et al. (2020). Mathematical modelling of alternative pathway of complement system. *Bulletin of mathematical biology*, 82(2):1–32.

Bansal, L., Nichols, E.-M., Howsmon, D. P., Neisen, J., Bessant, C., Cunningham, F., Petit-Frere, S., Ludbrook, S., and Damian, V. (2022). Mathematical modeling of complement pathway dynamics for target validation and selection of drug modalities for complement therapies. *Frontiers in pharmacology*, page 1112.

Carvelli, J., Demaria, O., Vély, F., Batista, L., Chouaki Benmansour, N., Fares, J., Carpentier, S., Thibult, M.-L., Morel, A., Remark, R., et al. (2020). Association of covid-19 inflammation with activation of the c5a–c5ar1 axis. *Nature*, 588(7836):146–150.

Chalise, J. P., Narendra, S. C., Paudyal, B. R., and Magnusson, M. (2013). Interferon alpha inhibits antigen-specific production of proinflammatory cytokines and enhances antigen-specific transforming growth factor beta production in antigen-induced arthritis. *Arthritis research & therapy*, 15(5):1–13.

Chen, Y., Chu, J. M. T., Chang, R. C. C., and Wong, G. T. C. (2022). The complement system in the central nervous system: From neurodevelopment to neurodegeneration. *Biomolecules*, 12(2):337.

Chouaki Benmansour, N., Carvelli, J., and Vivier, E. (2021). Complement cascade in severe forms of covid-19: Recent advances in therapy. *European Journal of Immunology*, 51(7):1652–1659.

Detsika, M. G. and Lianos, E. A. (2021). Regulation of complement activation by heme oxygenase-1 (ho-1) in kidney injury. *Antioxidants*, 10(1):60.

Dijkstra, D. J., Joeloemsingh, J. V., Bajema, I. M., and Trouw, L. A. (2019). Complement activation and regulation in rheumatic disease. In *Seminars in immunology*, volume 45, page 101339. Elsevier.

Dos Santos, R. S., Marroqui, L., Grieco, F. A., Marselli, L., Suleiman, M., Henz, S. R., Marchetti, P., Wernersson, R., and Eizirik, D. L. (2017). Protective role of complement c3 against cytokine-mediated β-cell apoptosis. *Endocrinology*, 158(8):2503–2521.

Fan, Y., Wang, Y., Yu, S., Chang, J., Yan, Y., Wang, Y., and Bian, Y. (2021). Natural products provide a new perspective for anti-complement treatment of severe covid-19: a review. *Chinese medicine*, 16(1):1–15.

Forneris, F., Wu, J., Xue, X., Ricklin, D., Lin, Z., Sfyroera, G., Tzekou, A., Volokhina, E., Granneman, J. C., Hauhart, R., et al. (2016). Regulators of complement activity mediate inhibitory mechanisms through a common c3b-binding mode. *The EMBO journal*, 35(10):1133–1149.

Garred, P., Tenner, A. J., and Mollnes, T. E. (2021). Therapeutic targeting of the complement system: from rare diseases to pandemics. *Pharmacological reviews*, 73(2):792–827.

Hembram, P. (2021). An outline of sars-cov-2 pathogenesis and the complement cascade of immune system. *Bulletin of the National Research Centre*, 45(1):1–10.

Henry, B. M., Szergyuk, I., Oliveira, M. H. S. d., Lippi, G., Benoit, J. L., Vikse, J., and Benoit, S. W. (2021). Complement levels at admission as a reflection of coronavirus disease 19 (covid-19) severity state. *Journal of Medical Virology*.

Hovland, A., Jonasson, L., Garred, P., Yndestad, A., Aukrust, P., Lappegård, K. T., Espevik, T., and Mollnes, T. E. (2015). The complement system and toll-like receptors as integrated players in the pathophysiology of atherosclerosis. *Atherosclerosis*, 241(2):480–494.

Jodele, S. and Köhl, J. (2021). Tackling covid-19 infection through complement-targeted immunotherapy. *British Journal of Pharmacology*, 178(14):2832–2848.

Kim, A. H., Wu, X., and Atkinson, J. P. (2020). The beneficial and pathogenic roles of complement in covid-19. *Cleveland Clinic journal of medicine*.

Kisserli, A., Schneider, N., Audonnet, S., Tabary, T., Goury, A., Cousson, J., Mahmoudi, R., Bani-Sadr, F., Kanagaratnam, L., Jolly, D., et al. (2021). Acquired decrease of the c3b/c4b receptor (cr1, cd35) and increased c4d deposits on erythrocytes from icu covid-19 patients. *Immunobiology*, 226(3):152093.

Lubbers, R., Van Essen, M., Van Kooten, C., and Trouw, L. (2017). Production of complement components by cells of the immune system. *Clinical & Experimental Immunology*, 188(2):183–194.

Ng, N. and Powell, C. A. (2021). Targeting the complement cascade in the pathophysiology of covid-19 disease. *Journal of Clinical Medicine*, 10(10):2188.

Noris, M., Benigni, A., and Remuzzi, G. (2020). The case of complement activation in covid-19 multiorgan impact. *Kidney international*, 98(2):314–322.

Paracha, R. Z., Ahmad, J., Ali, A., Hussain, R., Niazi, U., Tareen, S. H. K., and Aslam, B. (2014). Formal modelling of toll like receptor 4 and jak/stat signalling pathways: insight into the roles of socs-1, interferon-β and proinflammatory cytokines in sepsis. *PloS one*, 9(9):e108466.

Polycarpou, A., Howard, M., Farrar, C. A., Greenlaw, R., Fanelli, G., Wallis, R., Klavinskis, L. S., and Sacks, S. (2020). Rationale for targeting complement in covid-19. *EMBO molecular medicine*,

12(8):e12642.

Poppelaars, F., Faria, B., Gaya da Costa, M., Franssen, C. F., Van Son, W. J., Berger, S. P., Daha, M. R., and Seelen, M. A. (2018). The complement system in dialysis: a forgotten story? *Frontiers in immunology*, 9:71.

Ram Kumar Pandian, S., Arunachalam, S., Deepak, V., Kunjiappan, S., and Sundar, K. (2020). Targeting complement cascade: an alternative strategy for covid-19. *3 Biotech*, 10(11):1–10.

Risitano, A. M., Mastellos, D. C., Huber-Lang, M., Yancopoulou, D., Garlanda, C., Ciceri, F., and Lambris, J. D. (2020). Complement as a target in covid-19? *Nature Reviews Immunology*, 20(6):343–344.

Saeed, M. T., Ahmad, J., Baumbach, J., Pauling, J., Shafi, A., Paracha, R. Z., Hayat, A., and Ali, A. (2018). Parameter estimation of qualitative biological regulatory networks on high performance computing hardware. *BMC systems biology*, 12(1):1–15.

Shemesh, M., Aktepe, T. E., Deerain, J. M., McAuley, J. L., Audsley, M. D., David, C. T., Purcell, D. F., Urin, V., Hartmann, R., Moseley, G. W., et al. (2021). Sars-cov-2 suppresses ifnβ production mediated by nsp1, 5, 6, 15, orf6 and orf7b but does not suppress the effects of added interferon. *PLoS pathogens*, 17(8):e1009800.

Shibabaw, T., Molla, M. D., Teferi, B., and Ayelign, B. (2020). Role of ifn and complements system: Innate immunity in sars-cov-2. *Journal of Inflammation Research*, 13:507.

Shinjyo, N., Kagaya, W., and Pekna, M. (2021). Interaction between the complement system and infectious agents–a potential mechanistic link to neurodegeneration and dementia. *Frontiers in Cellular Neuroscience*, page 293.

Showers, C. R., Nuovo, G. J., Lakhanpal, A., Siegel, C. H., Aizer, J., Elreda, L., Halevi, A., Lai, A. R., Erkan, D., and Magro, C. M. (2021). A covid-19 patient with complement-mediated coagulopathy and severe thrombosis. *Pathobiology*, 88(1):14–22.

Thurman, J. M. and Renner, B. (2011). Dynamic control of the complement system by modulated expression of regulatory proteins. *Laboratory investigation*, 91(1):4–11.

Xie, C. B., Jane-Wit, D., and Pober, J. S. (2020). Complement membrane attack complex: new roles, mechanisms of action, and therapeutic targets. *The American journal of pathology*, 190(6):1138–1150.

Yan, B., Freiwald, T., Chauss, D., Wang, L., West, E., Mirabelli, C., Zhang, C. J., Nichols, E.-M., Malik, N., Gregory, R., et al. (2021). Sars-cov-2 drives jak1/2-dependent local complement hyperactivation. *Science Immunology*, 6(58).

Yang, L., Wang, J., Hui, P., Yarovinsky, T. O., Badeti, S., Pham, K., and Liu, C. (2021). Potential role of ifn-α in covid-19 patients and its underlying treatment options. *Applied Microbiology and Biotechnology*, pages 1–11.

Zewde, N., Gorham Jr, R. D., Dorado, A., and Morikis, D. (2016). Quantitative modeling of the alternative pathway of the complement system. *PloS one*, 11(3):e0152337.

Zhang, J., Zhao, C., and Zhao, W. (2021). Virus caused imbalance of type i ifn responses and inflammation in covid-19. *Frontiers in Immunology*, 12:1204.
