# Supplementary material for: Modelling and analysis of the complement system signalling pathways: roles of C3, C5a and pro-inflammatory cytokines in SARS-CoV-2 infection
Source: PeerJ. 2023 Sep 20;11:e15794. doi: 10.7717/peerj.15794 (PMC10517668; doi:10.7717/peerj.15794)
Supplement: Supplemental Information 25 [file peerj-11-15794-s025.pdf]

For normal condition BRN modelling: Entry of logical parameters set as tabulated in Table 2 in SMBioNet and GINsim tolls with description of the resources.

S.no	SMBioNet Kinetic parameters with description	GINsim kinetic parameters
1.	$K_{CoV2}\{\quad\} = 0$; PICyts and MAC are active for CoV2.	$K_{CoV2}\{PICyts, MAC\} = 0$
2.	$K_{CoV2}\{MAC\} = 0$; PICyts is active, MAC is inactive for CoV2.	$K_{CoV2}\{PICyts\} = 0$
3.	$K_{CoV2}\{PICyts\} = 0$; PICyts is inactive, MAC is active for CoV2.	$K_{CoV2}\{MAC\} = 0$
4.	$K_{CoV2}\{MAC,\ PICyts\} = 0$; MAC and PICyts are inactive for CoV2.	$K_{CoV2}\{\quad\} = 0$
5.	$K_{C3}\{\quad\} = 0$; CoV2 and PICyts are inactive and FI-CR1-DAF is active for C3.	$K_{C3}\{FI - CR1 - DAF\} = 0$
6.	$K_{C3}\{CoV2\} = 1$; CoV2 and FI-CR1-DAF are active, PICyts is inactive for C3.	$K_{C3}\{CoV2, \mathrm{FI - CR1 - DAF}\} = 1$
7.	$K_{C3}\{\mathrm{FI - CR1 - DAF}\} = 0$; FI-CR1-DAF, CoV2 and PICyts are inactive.	$K_{C3}\{\quad\} = 0$
8.	$K_{C3}\{\mathrm{PICyts}\} = 0$; PICyts, FI-CR1-DAF are active and CoV2 is inactive for C3.	$K_{C3}\{\mathrm{PICyts}, \mathrm{FI - CR1 - DAF}\} = 0$
9.	$K_{C3}\{CoV2, \mathrm{FI - CR1 - DAF}\} = 1$; CoV2 is active, FI-CR1-DAF and PICyts are inactive for C3	$K_{C3}\{CoV2\} = 1$
10.	$K_{C3}\{\mathrm{FI - CR1 - DAF},\ PICyts\} = 1$; PICyts is active, FI-CR1-DAF and CoV2 are inactive for C3.	$K_{C3}\{PICyts\} = 1$
11.	$K_{C3}\{\mathrm{CoV2},\ PICyts\} = 1$; CoV2, PICyts and FI-CR1-DAF are active for C3.	$K_{C3}\{\mathrm{CoV2}, PICyts, \mathrm{FI - CR1 - DAF}\} = 1$
12.	$K_{C3}\{\mathrm{CoV2},\ PICyts, \mathrm{FI - CR1 - DAF}\} = 1$; CoV2, PICyts are active and FI-CR1-DAF is inactive for C3.	$K_{C3}\{\mathrm{CoV2},\ PICyts\} = 1$
13.	$K_{C5a}\{\quad\} = 0$; C3 and PICyts are inactive and FI-CR1-DAF is active for C5a.	$K_{C5a}\{\mathrm{FI - CR1 - DAF}\} = 0$
14.	$K_{C5a}\{C3\} = 1$; C3, FI-CR1-DAF are active and PICyts is inactive for C5a.	$K_{C5a}\{C3, \mathrm{FI - CR1 - DAF}\} = 1$
15.	$K_{C5a}\{\mathrm{FI - CR1 - DAF}\} = 0$; FI-CR1-DAF, C3 and PICyts are inactive for C5a.	$K_{C5a}\{\quad\} = 0$
16.	$K_{C5a}\{PICyts\} = 0$; PICyts, FI-CR1-DAF are active and C3 is inactive for C5a.	$K_{C5a}\{\mathrm{PICyts},\ \mathrm{FI - CR1 - DAF}\} = 0$
17.	$K_{C5a}\{C3,\ FI - CR1 - DAF\} = 1$; C3 is active, FI-CR1-DAF and PICyts are inactive for C5a.	$K_{C5a}\{C3\} = 1$
18.	$K_{C5a}\{FI - CR1 - DAF, PICyts\} = 0$; FI-CR1-DAF, C3 are inactive, and PICyts active for C5a.	$K_{C5a}\{PICyts\} = 0$
19.	$K_{C5a}\{C3, PICyts\} = 1$; C3 and PICyts and FI-CR1-DAF are active for C5a.	$K_{C5a}\{C3, PICyts, \mathrm{FI - CR1 - DAF}\} = 1$
20.	$K_{C5a}\{C3, PICyts, \mathrm{FI - CR1 - DAF}\} = 1$; C3, PICyts are active and FI-CR1-DAF is inactive for C5a.	$K_{C5a}\{C3, PICyts\} = 1$

21.	$K_{MAC}\{\ \ \}=0$; C3 and PICyts are inactive for MAC.	$K_{MAC}\{\ \ \}=0$
22.	$K_{MAC}\{C3\}=1$; C3 is active and PICyts is inactive for MAC.	$K_{MAC}\{C3\}=1$
23.	$K_{MAC}\{PICyts\}=1$; PICyts is active and C3 is inactive for MAC.	$K_{MAC}\{PICyts\}$ =1
24.	$K_{MAC}\{C3, PICyts\}=1$; C3 and PICyts are active for MAC.	$K_{MAC}\{C3, PICyts\}=1$
25.	$K_{FI-CR1-DAF}\{\ \ \}=0$; PICyts is inactive for FI-CR1-DAF.	$K_{FI-CR1-DAF}\{\ \ \}=0$
26.	$K_{FI-CR1-DAF}\{PICyts\}=1$; PICyts is active for FI-CR1-DAF.	$K_{FI-CR1-DAF}\{PICyts\}=1$
27.	$K_{PICyts}\{\ \ \}=0$; C3, C5a are inactive and FI-CR1-DAF is active for PICyts.	$K_{PICyts}\{\mathrm{FI-CR1-DAF}\}=0$
28.	$K_{PICyts}\{C3\}=0$; C3, FI-CR1-DAF are active and C5a is inactive for PICyts.	$K_{PICyts}\{C3, FI-CR1-DAF\}=0$
29.	$K_{PICyts}\{C5a\}=2$; C5a, FI-CR1-DAF are active and C3 is inactive for PICyts.	$K_{PICyts}\{C5a, FI-CR1-DAF\ \}=2$
30.	$K_{PICyts}\{\mathrm{FI-CR1-DAF}\}=0$; FI-CR1-DAF, C3, and C5a are inactive for PICyts.	$K_{PICyts}\{\}=0$
31.	$K_{PICyts}\{\mathrm{C3, C5a}\}=2$; C3, C5a, and FI-CR1-DAF are active for PICyts.	$K_{PICyts}\{\mathrm{C3, C5a\ , FI-CR1-DAF}\}=2$
32.	$K_{PICyts}\{\mathrm{C5a, FI-CR1-DAF}\}=2$; C5a is active, FI-CR1-DAF and C3 are inactive for PICyts.	$K_{PICyts}\{\mathrm{C5a}\}=2$
33.	$K_{PICyts}\{\mathrm{C3, FI-CR1-DAF}\}=1$; C3 is active, FI-CR1-DAF and C5a are inactive for PICyts.	$K_{PICyts}\{\mathrm{C3}\}=1$
34.	$K_{PICyts}\{\mathrm{C3, C5a, FI-CR1-DAF}\}=2$; C3 and C5a are active, FI-CR1-DAF is inactive for PICyts.	$K_{PICyts}\{\mathrm{C3, C5a}\}$ =2
