# Supplementary material for: Modelling and analysis of the complement system signalling pathways: roles of C3, C5a and pro-inflammatory cytokines in SARS-CoV-2 infection
Source: PeerJ. 2023 Sep 20;11:e15794. doi: 10.7717/peerj.15794 (PMC10517668; doi:10.7717/peerj.15794)
Supplement: Supplemental Information 27 [file peerj-11-15794-s027.pdf]

Experimental observations	References
C3 level has been reported high in mild, moderate, and severe conditions.	(Henry et al., 2021; Ma et al., 2021; Showers et al., 2021; B. Yan et al., 2021)
The concentration level of complement peptide C5a has been reported to be elevated.	(Alosaimi et al., 2021; Busch et al., 2020; Kurtovic & Beeson, 2021; Ma et al., 2021; Marina Noris et al., 2020; Prendecki et al., 2020; Ram Kumar Pandian et al., 2020; Showers et al., 2021)
Expression level of terminal complement complex MAC is high in ICU admitted patients compared to general wards admissions	(Alosaimi et al., 2021; De Nooijer et al., 2021; Ma et al., 2021; Marina Noris et al., 2020; Showers et al., 2021)
Concentration of CR1 largely decreased in severely affected Patients.	(Kisserli et al., 2021)
Concentration Level of PICyts highly expressed in the form of cytokine storm.	(Alosaimi et al., 2021; García, 2020; Merad & Martin, 2020)
Lymphocyte counted in the severe and critical patients reported significantly low.	(Showers et al., 2021; W. Yan et al., 2021)
Level of negative regulatory protein FI has been identified significantly low.	(Alosaimi et al., 2021)
Leucocytes such as Inflammatory Cells (ICs) neutrophils, and macrophages counted to be high.	(Busch et al., 2020; S. Mueller-Ortiz et al., 2017; Ram Kumar Pandian et al., 2020; Showers et al., 2021; W. Yan et al., 2021)
Lower lymphocytes have been reported in CBC reports of COVID-19 patients.	(Kazancioglu et al., 2020)
Concentration level of IFNα and IFNβ have been reported low. It has been known that C5a suppressed IFNβ production.	(Contoli et al., 2021; S. L. Mueller-Ortiz et al., 2017)
Type 1 interferon IFNα and IFNβ treatment against SARSCoV2 significantly has reduced the viral titers.	(Mantlo et al., 2020)
Expression level of DAF reported high in COVID-19.	(Lage et al., 2022)

Table1. For SARS-CoV-2 infection experimentally observed concentration levels of different entities.

References

Alosaimi, B., Mubarak, A., Hamed, M. E., Almutairi, A. Z., Alrashed, A. A., AlJuryyan, A., Enani, M., Alenzi, F. Q., & Alturaiki, W. (2021). Complement Anaphylatoxins and Inflammatory Cytokines as Prognostic Markers for COVID-19 Severity and In-Hospital Mortality. *Frontiers in Immunology*, *12*(July), 1–13. https://doi.org/10.3389/fimmu.2021.668725

Busch, M. H., Timmermans, S. A. M. E. G., Nagy, M., Visser, M., Huckriede, J., Aendekerk, J. P., De Vries, F., Potjewijd, J., Jallah, B., Ysermans, R., Oude Lashof, A. M. L., Breedveld, P. H., Van De Poll, M. C. G., Van De Horst, I. C. C., Van Bussel, B. C. T., Theunissen, R. O. M. F. I. H., Spronk, H. M. H., Damoiseaux, J. G. M. C., Ten Cate, H., … Van Paassen, P. (2020). Neutrophils and Contact Activation of Coagulation as Potential Drivers of COVID-19. *Circulation*, *142*(18), 1787–1790. https://doi.org/10.1161/CIRCULATIONAHA.120.050656

Contoli, M., Papi, A., Tomassetti, L., Rizzo, P., Vieceli Dalla Sega, F., Fortini, F., Torsani, F., Morandi, L., Ronzoni, L., Zucchetti, O., Pavasini, R., Fogagnolo, A., Volta, C. A., Bartlett, N. W., Johnston, S. L., Spadaro, S., & Campo, G. (2021). Blood Interferon-α Levels and Severity, Outcomes, and Inflammatory Profiles in Hospitalized COVID-19 Patients. *Frontiers in Immunology*, *12*(March), 1–10. https://doi.org/10.3389/fimmu.2021.648004

De Nooijer, A. H., Grondman, I., Janssen, N. A. F., Netea, M. G., Willems, L., Van De Veerdonk, F. L., Giamarellos-Bourboulis, E. J., Toonen, E. J. M., Joosten, L. A. B., Jaeger, M., Dijkstra, H., Lemmers, H., Van Emst, L., Schraa, K., Jacobs, C., Hijmans, A., Jansen, T., Weren, F., Fransman, L., … Klop-Riehl, M. (2021). Complement Activation in the Disease Course of Coronavirus Disease 2019 and Its Effects on Clinical Outcomes. *Journal of Infectious Diseases*, *223*(2), 214–224. https://doi.org/10.1093/infdis/jiaa646

García, L. F. (2020). Immune Response, Inflammation, and the Clinical Spectrum of COVID-19. *Frontiers in Immunology*, *11*(June), 4–8. https://doi.org/10.3389/fimmu.2020.01441

Henry, B. M., Szergyuk, I., de Oliveira, M. H. S., Lippi, G., Benoit, J. L., Vikse, J., & Benoit, S. W. (2021). Complement levels at admission as a reflection of coronavirus disease 2019 (COVID-19) severity state. *Journal of Medical Virology*, *93*(9), 5515–5522. https://doi.org/10.1002/jmv.27077

Kazancioglu, S., Bastug, A., Ozbay, B. O., Kemirtlek, N., & Bodur, H. (2020). The Role of Hematological Parameters in Patients with Coronavirus Disease 2019 and Influenza Virus Infection. *Epidemiology and Infection*. https://doi.org/10.1017/S095026882000271X

Kisserli, A., Schneider, N., Audonnet, S., Tabary, T., Goury, A., Cousson, J., Mahmoudi, R., Bani-Sadrf, F., Kanagaratnam, L., Jolly,

D., & Cohen, J. H. (2021). Acquired decrease of the C3b/C4b receptor (CR1, CD35) and increased C4d deposits on erythrocytes from ICU COVID-19 patients. *Immunobiology*, *226*(3). https://doi.org/https://doi.org/10.1016%2Fj.imbio.2021.152093

Kurtovic, L., & Beeson, J. G. (2021). Complement Factors in COVID-19 Therapeutics and Vaccines. *Trends in Immunology*, *42*(2), 94–103. https://doi.org/10.1016/j.it.2020.12.002

Lage, S. L., Rocco, J. M., Laidlaw, E., Rupert, A., Galindo, F., Kellogg, A., Kumar, P., Poon, R., Wortmann, G. W., Lisco, A., Manion, M., & Sereti, I. (2022). Activation of Complement Components on Circulating Blood Monocytes From COVID-19 Patients. *Frontiers in Immunology*, *13*(February), 1–9. https://doi.org/10.3389/fimmu.2022.815833

Ma, L., Sahu, S. K., Cano, M., Kuppuswamy, V., Bajwa, J., McPhatter, J., Pine, A., Meizlish, M. L., Goshua, G., Chang, C. H., Zhang, H., Price, C., Bahel, P., Rinder, H., Lei, T., Day, A., Reynolds, D., Wu, X., Schriefer, R., … Kulkarni, H. S. (2021). Increased complement activation is a distinctive feature of severe SARS-CoV-2 infection. *Science Immunology*, *6*(59), 1–17. https://doi.org/10.1126/sciimmunol.abh2259

Mantlo, E., Bukreyeva, N., Maruyama, J., & Paessler, S. (2020). Antiviral activities of type I interferons to SARS-CoV-2 infection. *Antivial Research*, *179*. https://doi.org/10.1016/j.antiviral.2020.104811.

Marina Noris, Ariela Benigni, & Giuseppe Remuzzi1. (2020). The case of complement activation in COVID-19 multiorgan impact. *Kidney International.*, *98*(2), 314–322. https://doi.org/10.1016/%0Aj.kint.2020.05.013

Merad, M., & Martin, J. C. (2020). Pathological inflammation in patients with COVID-19: a key role for monocytes and macrophages. *Nature Reviews Immunology*, *20*(6), 355–362. https://doi.org/10.1038/s41577-020-0331-4

Mueller-Ortiz, S. L., Daniel G. Calame, N. S., Li, Y.-D., & Wetsel, R. A. (2017). The Complement Anaphylatoxins, C5a and C3a, Suppress Interferon-Beta Production in Response to Listeria monocytogenes by Inhibition of the Cyclic Dinucleotide-Activated Cytosolic Surveillance Pathway. *Immunol*, *198*(8), 3237–3244. https://doi.org/10.4049/jimmunol.1601420

Mueller-Ortiz, S., L., T., Calame, D. G., Shenoi, N., Li, Y.-D., & Wetsel, R. A. (2017). Cytokine storm in COVID-19: pathogenesis and overview of anti-inflammatory agents used in treatment. *Clinical Rheumatology*, *198*(8), 3237–3244. https://doi.org/doi:10.4049/jimmunol.1601420.

Prendecki, M., Clarke, C., Medjeral-Thomas, N., McAdoo, S. P., Sandhu, E., Peters, J. E., Thomas, D. C., Willicombe, M., Botto, M., & Pickering, M. C. (2020). Temporal changes in complement activation in haemodialysis patients with COVID-19 as a predictor of disease progression. *Clinical Kidney Journal*, *13*(5), 889–896. https://doi.org/10.1093/CKJ/SFAA192

Ram Kumar Pandian, S., Arunachalam, S., Deepak, V., Kunjiappan, S., & Sundar, K. (2020). Targeting complement cascade: an alternative strategy for COVID-19. *3 Biotech*, *10*(11), 1–10. https://doi.org/10.1007/s13205-020-02464-2

Showers, C. R., Nuovo, G. J., Lakhanpal, A., Siegel, C. H., Aizer, J., Elreda, L., Halevi, A., Lai, A. R., Erkan, D., & Magro, C. M. (2021). A Covid-19 Patient with Complement-Mediated Coagulopathy and Severe Thrombosis. *Pathobiology*, *88*(1), 28–36. https://doi.org/10.1159/000512503

Yan, B., Freiwald, T., Chauss, D., Wang, L., West, E., Mirabelli, C., Zhang, C. J., Nichols, E. M., Malik, N., Gregory, R., Bantscheff, M., Ghidelli-Disse, S., Kolev, M., Frum, T., Spence, J. R., Sexton, J. Z., Alysandratos, K. D., Kotton, D. N., Pittaluga, S., … Kazemian, M. (2021). SARS-CoV-2 drives JAK1/2-dependent local complement hyperactivation. *Science Immunology*, *6*(58), 1–19. https://doi.org/10.1126/sciimmunol.abg0833

Yan, W., Chen, D., Bigambo, F. M., Wei, H., Wang, X., & Xia, Y. (2021). Differences of blood cells, lymphocyte subsets and cytokines in COVID-19 patients with different clinical stages: a network meta-analysis. *BMC Infectious Diseases*, *21*(1), 1–9. https://doi.org/10.1186/s12879-021-05847-9
