# Supplementary material for: Modelling and analysis of the complement system signalling pathways: roles of C3, C5a and pro-inflammatory cytokines in SARS-CoV-2 infection
Source: PeerJ. 2023 Sep 20;11:e15794. doi: 10.7717/peerj.15794 (PMC10517668; doi:10.7717/peerj.15794)
Supplement: Supplemental Information 11 [file peerj-11-15794-s011.pdf]

Modelling and analysis of complement system signalling pathways: Roles of C3, C5a and pro-inflammatory cytokines in SARS-CoV-2 infection

Didar Murad[1], Rehan Zafar Paracha[1*], Muhammad Tariq Saeed[1], Jamil Ahmad[2], Ammar Mushtaq[1], and Maleeha Humayun[1]

[1]**School of Interdisciplinary Engineering and Sciences/Department of Sciences/National University of Sciences and Technology, H-12, Islamabad, Pakistan**
[2]**Department of Computer Science and Information Technology/University of Malakand, Chakdara, Khyber Pakhtunkhwa, Pakistan**

Corresponding author:
Rehan Zafar Paracha[1*]

Email address: rehan@sines.nust.edu.pk

Semantics of Asynchronous multi-valued Kinetic Logic Formalism (Paracha et al., 2014; Saeed et al., 2016)

Definition 1 (Biological Regulatory Network)

A BRN is a labeled directed graph D = (V, E) where V=$\{p_1,...,p_n\}$ is a set of n vertices or nodes representing biological entities (proteins or genes) and $E = \{(p_i, p_j) \mid (p_i, p_j) \in V \times V\}$ is a set of edges representing interactions. An edge is labeled with an integer α (representing thresholds of interactions) and β representing $''+''$ or $''-''$ signs (for activation or inhibition). In the toy example of Figure 1A, V=$\{p_1, p_2, p_3\}$ and E = $\{(p_1,\ p_2),\ (p_2,\ p_3),\ (p_3,\ p_1)\}$.
We denote the successors of an entity $p_i \in V$ by the set $S^+(p_i)$ and its predecessors by the set $S^-(p_i)$. For example, $S^+(p_1) = \{p_2\}, S^-(p_1) = \{p_3\}$. The number of target entities of p_i is $l_{p_i} =\mid S^+(p_i) \mid$ which represents the out degree of p_i. The threshold levels of entity say p_1 is set within the range $''1''$ to $''$ total number of outgoing edges $''$. As p_1 has only one outgoing edge towards p_2, the only threshold value is set as $''1''$. Let $F_{p_i} = \{0, 1, 2, \ldots, k_{p_i}\}$ where, $k_{p_i} \leq l_{p_i}$ is the set of abstract expression of each entry p_i.

Definition 2 (Activation and Inhibition)

Suppose p_1, p_2, and p_3 are three entities shown in Figure 1A, threshold level of any entity is denoted by θ. If p_1 increases rate of activation of p_2, then p_1 is an activator (upregulator) of p_2. In this case, always concentration level of p_1 increases or decreases, it will affect concentration level of p_2 in similar manner. This relationship is shown by dummy tendency graphs as sigmoidal curves (Figure 1 D). When p_1 is below θ, it slightly changes the synthesis rate of p_2. When p_1 reaches at θ activation rate of p_2 rapidly increases up to the limit of concentration level assumed shown in Figure 1 D.
If p_3 decreases activation rate of p_1, then p_3 is known as inhibitor (downregulator) of p_1. Downregulation mechanism also depends on θ of p_3. When concentration level of p_3 reaches at θ then p_3 will be efficient in lowering p_1 production levels up to a level where p_1 can be totally deactivated. The affect of inhibition is also of sigmoidal nature.

Definition 3 (Qualitative State)

A qualitative state is an n-tuple vector $\{q_{p_i}$ for i=1 to n$\}$ such that $q_{p_i} \in F_{p_i}$ represent abstract concentration level of p_i. Basically, qualitative state indicates a configuration of all the components of a BRN. In a

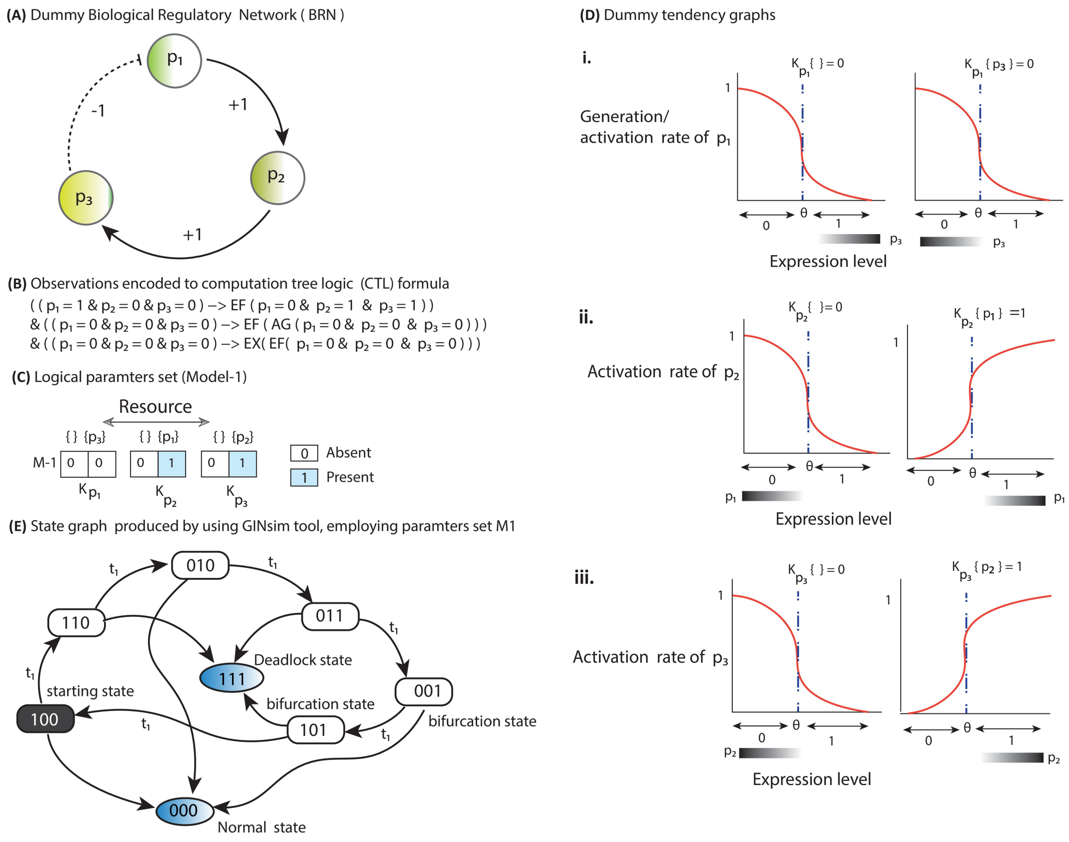

Figure 1. The schematic of the qualitative modelling based on the Asynchronous Boolean logic approach. (A) A toy BRN indicating a directed graph comprises of three entities p_1, p_2, and p_3. The edges p_1 to p_2, p_2 to p_3, and p_3 to p_1 indicating entities interaction. The edges are labeled with positive and negative signs representing activation and inhibition with threshold level 1 (see definition 1 and definition 2). (B) Let assumed observations that are encoded to CTL formula. (C) Using model checker NuSMV in SMBioNet tool (File S16), a parameters set (model-1) M-1 is generated. The CTL model checking infers logical parameters for the BRN which capture asynchronous evolution of entities and verify the CTL formula. (D) Dummy tendency graphs show generation level of targeted entity due to presence or absence of resource entity as activator or inhibitor. (E) Using definition 5, BRN is simulated to state graph shows that all the expression levels (states) of entities evolve in asynchronous fashion i.e. in the state only one entity changes its states. The state graph comprises of trajectory (t_1) indicates SCC (definition 6) attractors as bistability deadlock state $(1,1,1)$ and normal state $(0,0,0)$. Bifurcation states $(0,0,1)$ and $(1,0,1)$ from where the state transitions can evolve either in the direction of deadlock state or recovery state.

given state, each node p_i as a target is regulated by its predecessors $S^{-}(p_i)$ formally specified by set of resources defined below.

Definition 4 (Set of Resources)
At a given level of expression q_{p_y} for entry $p_y \in V$ the set of resources is defined as: $M_{(p_y)}$=$\{p_x \in S^{-}(p_y) \mid (q_{p_x} \geq \alpha_{p_x,p_y}$ & $\beta_{p_x,p_y} = ''+'')$ or $(q_{p_x} < \alpha_{p_x,p_y}$ & $\beta_{p_x,p_y} = ''-'')\}$.
It is to be noted that presence of activators or absence of inhibitors is assumed as resource. $M_{(p_i)}$ can include activators and inhibitors of p_i. The targets toward which the concentration levels of p_i evolve, depend on set of positive integers $K_{p_i}(M_{(p_i)})$ known as logical parameters, indexed by $M_{(p_i)}$.
The logical parameter $K_{p_i}(M_{(p_i)})$ at an expression level q_{p_i} of p_i provides information about evolution of entity p_i, such that

$$q_{p_i} \Rsh K_{p_i}(M_{(p_i)}) = \left\{ \begin{array}{c} q_{p_i}+1 \quad if \quad q_{p_i} < K_{p_i}(M_{(p_i)}) \\ q_{p_i}-1 \quad if \quad q_{p_i} > K_{p_i}(M_{(p_i)}) \\ q_{p_i} \quad if \quad q_{p_i} = K_{p_i}(M_{(p_i)}) \end{array} \right\} \tag{1}$$

where: $q_{p_i} \quad \& \quad K_{p_i}(M_{(p_i)}) \in F_{p_i}$.
It illustrates the evolution from one level to another, where " $\Rsh$ " represents the evolution operator.

Definition 5 (State Graph/Space)
Consider the BRN $D = (V,E)$, for an entity say p_i, q_{p_i} represents its concentration level in a state $q \in Q$. Then state graph of BRN is a directed graph represented by $T = (Q,R)$, $R \subseteq$ Q $\times$ Q is a transition relation between states and $q \rightarrow q' \in$ R $\iff$

- $\exists$ unique u $\in$ V such that (s.t) $q_{p_u} \neq q'_{p_u}$ and $q'_{p_u} = q_{p_u} \Rsh K_{p_u}(M_{p_u})$, and
- $\forall$ v $\in$ V$\setminus\{$u$\}$ s.t $q'_{p_v} = q_{p_v}$.

Definition 6 (Strongly Connected Component)
Strongly connected component (SCC) is a portion of state graph $T = (Q,R)$ in which there is a trajectory from each state to another state. It define the regulatory cycle of signalling networks in which the system loop through specific trajectories. For example, let x and y are any qualitative states in T, directed paths from x to y and y to x constitute SCC. In State graph trajectories labelled with t_1 as $(1,0,0) \rightarrow (1,1,0) \rightarrow (0,1,0) \rightarrow (0,1,1) \rightarrow (0,0,1) \rightarrow (1,0,1) \rightarrow (1,0,0)$ represent SCC is shown in Figure 1E.

Definition 7 (Betweenness Centrality)
For the interaction graph $D = (V,E)$ with state graph $T = (Q,R)$, let x, y and z are any qualitative states in T, such that $x \neq y \neq$ z. Betweenness Centrality (BC) of state z is mathematically defined as:

$$C_b(z) = \sum_{(x,y)\in\Delta} \frac{\phi_{(x,y)}(z)}{\phi_{(x,y)}} \tag{2}$$

Where, $\phi_{(x,y)}(z)$ is total number of trajectories from x to y pass though z, $\phi_{(x,y)}$ is total number of short trajectories from x to y and Δ is the set of all order pairs (x,y).

REFERENCES

Paracha, R. Z., Ahmad, J., Ali, A., Hussain, R., Niazi, U., Tareen, S. H. K., and Aslam, B. (2014). Formal modelling of toll like receptor 4 and jak/stat signalling pathways: insight into the roles of socs-1, interferon-β and proinflammatory cytokines in sepsis. *PloS one*, 9(9):e108466.

Saeed, M. T., Ahmad, J., Kanwal, S., Holowatyj, A. N., Sheikh, I. A., Paracha, R. Z., Shafi, A., Siddiqa, A., Bibi, Z., Khan, M., et al. (2016). Formal modeling and analysis of the hexosamine biosynthetic pathway: role of o-linked n-acetylglucosamine transferase in oncogenesis and cancer progression. *PeerJ*, 4:e2348.
